# Early-Life Stress, HPA Axis Adaptation, and Mechanisms Contributing to Later Health Outcomes

**DOI:** 10.3389/fendo.2014.00073

**Published:** 2014-05-13

**Authors:** Jayanthi Maniam, Christopher Antoniadis, Margaret J. Morris

**Affiliations:** ^1^Department of Pharmacology, School of Medical Sciences, UNSW Australia, Sydney, NSW, Australia

**Keywords:** early-life stress, metabolic disorders, 11-beta hydroxysteroid dehydrogenase 1, hyperinsulinemia, liver, insulin signaling, glucocorticoids

## Abstract

Stress activates the hypothalamic–pituitary–adrenal (HPA) axis, which then modulates the degree of adaptation and response to a later stressor. It is known that early-life stress can impact on later health but less is known about how early-life stress impairs HPA axis activity, contributing to maladaptation of the stress–response system. Early-life stress exposure (either prenatally or in the early postnatal period) can impact developmental pathways resulting in lasting structural and regulatory changes that predispose to adulthood disease. Epidemiological, clinical, and experimental studies have demonstrated that early-life stress produces long term hyper-responsiveness to stress with exaggerated circulating glucocorticoids, and enhanced anxiety and depression-like behaviors. Recently, evidence has emerged on early-life stress-induced metabolic derangements, for example hyperinsulinemia and altered insulin sensitivity on exposure to a high energy diet later in life. This draws our attention to the contribution of later environment to disease vulnerability. Early-life stress can alter the expression of genes in peripheral tissues, such as the glucocorticoid receptor and 11-beta hydroxysteroid dehydrogenase (11β-HSD1). We propose that interactions between altered HPA axis activity and liver 11β-HSD1 modulates both tissue and circulating glucocorticoid availability, with adverse metabolic consequences. This review discusses the potential mechanisms underlying early-life stress-induced maladaptation of the HPA axis, and its subsequent effects on energy utilization and expenditure. The effects of positive later environments as a means of ameliorating early-life stress-induced health deficits, and proposed mechanisms underpinning the interaction between early-life stress and subsequent detrimental environmental exposures on metabolic risk will be outlined. Limitations in current methodology linking early-life stress and later health outcomes will also be addressed.

## Introduction

Stress can be defined as any condition including an adverse environment, experience, or perceived threat to alter an organism’s homeostasis, which elicits a physiological response involving both peripheral and central systems via the release of glucocorticoids from the adrenal cortex through activation of the hypothalamic–pituitary–adrenal (HPA) axis (1). Glucocorticoids serve as the critical end product of the HPA axis and negative feedback through glucocorticoid–glucocorticoid receptor binding in the hippocampus promotes adaptation and recovery from stress (2). Activity of the HPA axis plays a critical role in restoring homeostasis following imminent or acute stressor exposure (1). In contrast, recurrent or persistent activation of the HPA axis and the autonomic nervous system are associated with adverse health outcomes (3). Individuals respond differently to stressors, which can reflect a wide range of adversities in life from major events to daily conflicts and pressures. Increased levels of glucocorticoids interfere with energy utilization and modify metabolic hormones such as insulin and glucose, which are key regulators of energy metabolism (4–6). The elicited response affects multiple physiological systems including neuroendocrine, autonomic, and the immune system and is an established risk factor for the development of disease (7–10).

There is longstanding recognition of the impact of stress during critical early developmental periods such as childhood and the consequential association with adverse mental health outcomes and changes in brain development (11–13), however, less is known regarding how this dysfunction may confer increased metabolic disease risk. Emerging epidemiological evidence demonstrates that adverse early-life stress-induced dysregulation of the HPA axis and increases vulnerability for metabolic disorders. In particular, exposure to an adverse environment during prenatal and postnatal periods, such as lack of nutrition or starvation during war, traumatic experiences including childhood physical or sexual abuse, neglect, adverse parenting or medical trauma has been demonstrated to be one of the major risk factors contributing to the development metabolic disorders including insulin resistance, type 2 diabetes mellitus (T2DM), and hyperlipidemia (14–16).

While studies aimed at exploring underlying mechanisms are difficult to achieve in humans, animal studies have shed some light into the effects of early-life stress induced during the prenatal and postnatal periods on metabolic hormones and on peripheral tissues involved in glucose/insulin and lipid metabolism (17–24). In addition, the morphology of the pancreas is also affected by prenatal stress, with reduced beta-cell numbers (25). Thus, stress during early development may incur permanent alterations in morphology and function of key peripheral organs involved in metabolism of insulin, glucose and lipids, and these changes suggest programing effects of early-life stress (26).

This review will unravel how early-life stress induces metabolic derangements; an area that is less well explored. Despite the emergence of human data in the field, mechanisms elucidating the association between stress and adverse metabolic outcomes are lacking. Animal studies permit closer investigation of the stress system and through various manipulations, allow the mechanisms underlying effects of stress on metabolism to be explored. Of particular focus is exploring outcomes of stress during the perinatal and early-life period for later life metabolic outcomes.

## Does Early-Life Stress Affect Risk for Later Life Metabolic Deficit?

### Modeling early-life stress in animals

As direct examination of the prospective effects of early-life stress is not feasible in humans this had led to the development of numerous animal models to explore the question of whether early-life stress can affect risk for later life metabolic deficit. Animal models of early-life stress allow for controlled environmental manipulation throughout developmental periods and later life. With necessary caution these models can assist our understanding of the link between developmental and environment experiences and the conferred later life metabolic disease vulnerabilities. Inherent differences in human and rodent biological maturation and neuroendocrine development must be considered in study design and translation to human health. Humans give birth to mature young; with the final trimester of pregnancy being a period of rapid brain development (27, 28). Rodent offspring are born relatively immature with maximal growth phase initiated early in the postnatal period. Despite greater maturity of human offspring at the time of birth, development is far from complete with changes in neurological processes, synaptogenesis, synaptic pruning, and plastic changes in key functional areas including the hippocampus occurring until late adolescence (29, 30). The environment has been shown to impact this ongoing development beyond gestation. Children physically healthy at the time of birth who were abused in early life were shown to have reduced brain volume, correlated to age of abuse onset, and duration of the stress (31). Imposed stressors during rodent gestational or early postnatal life are suggested to model the period of gestation, early postnatal, or infancy in humans.

Humans and rodents have a vital dependence on adequate nourishment and care to ensure normal development. This altricial nature and vulnerable perinatal period across both species means that models of maternal care can provide insight into how early support and sensitivity to offspring needs can impact development. Changes in maternal care have been shown to impact rodent development with mother–offspring interactions such as licking and feeding providing critical input for normal neurobiological development and HPA axis function (32, 33). Adequate maternal contact during this period assists in maintaining rodents in their early-life hypo-responsive stress state and adverse experience through physical or psychological means or synthetic glucocorticoid administration can permanently alter HPA axis function (34, 35).

Three popular paradigms of postnatal early-life stress are maternal separation for varied periods of time from 15 min to 8 h, maternal deprivation (absence of the dam for a more extended period) and provision of only limiting nesting material. The maternal separation model has been studied for more than five decades, and is demonstrated in both mice and rats to affect the HPA axis, and behavioral responses in mothers and offspring in a sex dependant manner (36–39). Maternal deprivation is another common form of early-life stress which has been studied over decades (40–44). Twenty-four hours of maternal deprivation in neonatal rodents induced marked elevations in plasma corticosterone and decreases in plasma glucose and leptin, amongst other hormonal and neurotrophic factor changes (40, 43). This model, however appears to reflect a severe nutritional insult rather than psychological disturbances. Therefore, whether maternal deprivation represents adverse early experience in the human context is debatable. The limiting nesting paradigm, a more recently developed model of early-life stress, has been described elsewhere (35) but, briefly, involves limiting the dam’s available material for nest building, resulting in rudimentary and inadequate housing for offspring, a chronic stressor for the dam and pups. Limited nesting (LN) material, attempts to enhance commonalities to the human condition of childhood neglect and maternal stress in which the mother is present, yet care is abnormal and fragmented. Notably, the LN model has been demonstrated to impair HPA axis activity and induce behavioral deficits both in the dam and pups (35, 45, 46).

Recently, early-life stress models in non-human primates have demonstrated a lasting health impact following adverse early experiences. Altering secure attachment relationships during early life in Rhesus macaques significantly elevated prevalence and frequency of illness and increased bodyweight trajectory (47). Stress induced via variable foraging demand in bonnet macaques during lactation affected the metabolic profile of their offspring; a decreased glucose disposal rate was observed during hyperinsulinemic-euglycemic clamps in those exposed to early-life stress (48).

### Observations in human studies

Retrospective and observational studies in humans demonstrate that early-life experiences can influence later life metabolic outcome. Environmental changes during gestation and the early postnatal period may impact development and predict metabolic health outcomes. Manipulations of the early environment can affect the developing nervous system, shaping individual differences in physiological and behavioral responses to environmental insults. For example, disruption of the mother–infant relationship during early life contributes to neuroendocrine, neurochemical, and behavioral changes in the adult organism (49). Experience of adversity during early life and adolescence in the form of parental conflict or parental separation increased the risk of later life obesity (50). Similarly, experience of a range of early-life stressors was positively correlated with increased adult BMI in men, independently of mental health condition (51). These adverse early-life experiences are associated with persisting changes in HPA axis function in adult life with changes in the normal dynamics of the stress system and its end-point hormone cortisol (52). Commonly, a flattened cortisol circadian rhythm and hypo- or hyper-responsiveness to future novel stressors is observed (53, 54).

Barker’s theory postulates that low birth weight predicts increased disease risk later in life, including metabolic vulnerability (55). Given this association, identifying factors that influence gestational growth, and ultimately determine birth weight is important. Reduced birth weight and preterm birth have both been associated with psychosocial stress exposure during pregnancy (56–58). Having a low birth weight baby (<2500 g) was associated with stress-related psychiatric illness in pregnant mothers, such as melancholic depression (59). Prenatal psychological stress such as experiencing bereavement during pregnancy led to an increased risk of developing T2DM in their children during adulthood (60). Degree of social support during pregnancy has also been associated with birth weight (61). Other psychological stressors such as financial, relationship problems, and illness during pregnancy led to elevated glucose, insulin, and C-peptide levels during glucose tolerance test in their children aged 25 (62). Nutritional stress during the neonatal period has also been shown to adversely impact offspring health outcome, as explored below.

Famine exposure during pregnancy is a chronic early-life stress that is also known to affect birth weight and increase risk for metabolic disorders later in life. A well-documented period providing epidemiological evidence linking early-life adversity and health outcomes is the 1944–1945 Dutch Famine (see Table 1). A cohort of 741 subjects exposed to the Dutch Famine prenatally had a reduced birth weight, yet at adulthood these subjects had increased body weight, BMI, fasting proinsulin levels, and glucose intolerance (63). A report of 7557 women exposed to the Dutch famine showed increased risk for T2DM development in their offspring (64). Exposure to the Chinese famine during the 1960s showed similar adverse outcomes for offspring, with women having higher prevalence of metabolic disorders such as diabetes, hypertriglyceridemia, and hypertension (65). A cross-sectional study of subjects exposed to the Biafran famine during the Nigerian Civil War showed derangements in their metabolic profile during adulthood with increased risk for diabetes in both middle-aged men and non-pregnant women (66) (see Table 1). These data highlight that adverse early experiences, whether psychological or nutritional in nature during vulnerable developmental periods can impact offspring insulin and glucose metabolism during adulthood (Table 1).

**Table 1:** **Human early-life stress studies exploring metabolic outcomes**.

Early-life stressor	Participants	Offspring age	Exclusion criteria	Metabolic impact on offspring	Reference
*Prenatal*: maternal stress, holocaust exposure	137 adults, 74% reported parental holocaust exposure. Remainder considered unexposed controls	Middle-aged men and women	Psychosis, bipolar disorder, substance dependence	↑ Reported use of medications, including psychotropic, antihypertensives, dyslipidemia medication	Flory et al. (67)
			Organic mental disorder	↑ Association with having two or more metabolic syndrome components, e.g., T2DM, hypertension, dyslipidemia or increased BMI	
			Dementia; oral corticosteroids	
*Prenatal*: maternal stress, psychosocial	58 offspring, of whom 36 exposed to maternal stress. Remaining 22 considered unexposed controls	Young adults	Pregnancy complication Smoker Acute or chronic health problems	↑ BMI	Entringer et al. (62)
				↑ Very low-density lipoprotein (138%)	
				↓ High-density lipoprotein (16%) and low-density lipoprotein (33%)	
				*OGTT: offspring of mothers whom experienced psychosocial stress compared to control*	
				↑ Fasting plasma insulin levels (58%)	
				↑ Plasma insulin 2-h post-oral glucose load (59%)	
				↑ C-peptide 2-h post-oral glucose load (40%)	
*Prenatal*: maternal stress, natural disaster exposure	111 Women pregnant during or conceived within 3 months of the Quebec ice storm	Children, 5.5 years of age		↑ Obesity risk of offspring at 5.5 years old, associated with severity of objective maternal stress	Dancause et al. (68)
				Controlled for SES, pregnancy complications, breastfeeding, smoking, psychological function, and BMI	
*Prenatal*: maternal stress, natural disaster exposure	176 women pregnant during or conceived within 1 month of 1998 Quebec ice storm and their children	Children, mean age 13.5 years		Objective hardship positively correlated with insulin secretion (*P* < 0.01) and BMI (*P* < 0.02)	Dancause et al. (69)
*Prenatal*: maternal stress, bereavement	1,878,246 people, of whom 45,302 were exposed to stress. Remaining considered unexposed controls	Offspring followed for 2–32 years		↑ Risk for T2DM Second trimester identified as the most sensitive	Li et al. (60)
*Prenatal and postnatal*: maternal stress, famine	741 people born in Amsterdam before, during or after Dutch famine	Middle-aged men and women	Missing birth records	↑ Bodyweight, BMI and waist circumference in women 50 years of age exposed to early gestation famine vs. non-exposed controls	Ravelli et al. (70)
			Preterm birth (<37 weeks)	
			Deceased	
			Emigrated	
*Prenatal and postnatal*: maternal stress, famine	702 people born in Amsterdam before, during or after Dutch famine	Middle-aged men and women	Missing birth records	*OGTT: offspring exposed to famine compared to control*	Ravelli et al. (63)
			Preterm birth (<37 weeks)	↑ Fasting proinsulin levels and 2-h glucose concentrations	
			Diabetes	More pronounced if famine occurred during late gestation or with later life obesity	
			Deceased	
			Emigrated	
*Prenatal and postnatal*: maternal perceived stress	152 women surveyed during pregnancy/first year of offspring life, predominantly low-income population	Infants		↑ Risk of infant being overweight (*P* = 0.020)	Watt et al. (71)
				Correlation with consumption of sugar-sweetened beverages (*P* = 0.004) and with feeding infants sugar-sweetened beverages (*P* = 0.031)	
*Prenatal and postnatal*: maternal stress, depression	1249 women, depressive symptoms assessed during pregnancy and postpartum	Children 3 years of age	Multiple gestation	*Antenatal depression*	Ertel et al. (72)
			Issues with English	Smaller body size	
			Move prior to delivery	↑ Central adiposity	
			Gestational age greater than 22 weeks at first prenatal visit	*Postpartum depression*	
				↑ Overall adiposity	
				Independent of SES, BMI, and health condition during pregnancy	
*Postnatal*: childhood stress, death of a parent	135 bariatric surgery candidates	Middle-aged men and women	Substance abuse	↑ Risk of metabolic syndrome following childhood parental loss (*P* = 0.012)	Alciati et al. (73)
			Severe personality disorder	
			Mental retardation	
*Postnatal:* maternal stress (mental, physical, financial family structure) and altered food security	841 Children across 425 low-income households	Children, 3–17 years old	Households above 200% of poverty line	↑ Risk of offspring 3–10 years old being overweight or obese in food secure environments compared to periods of food insecurity (43.7%)	Gundersen et al. (74)
*Postnatal*: childhood maltreatment	67,853 women in Nurses Health Study II	25–42		Dose–response association between child physical and sexual abuse with adult T2DM. Hazard ratio for diabetes in child exposed to mild, moderate and severe are 1.03, 1.26 and 1.54 respectively	Rich-Edwards et al. (75)
*Postnatal*: childhood maltreatment	*n* = 972, born in between April 1972 and March 1973	32 years	Individuals with plasma c-reactive protein >10 mg/l	↑ Inflammation assessed by c-reactive protein	Danese et al. (76)
*Postnatal*: childhood maltreatment	342 from study of women health across the nation (SWAN)	45.7 year (mean age)		Physical abuse was associated with increased plasma triglyceride and blood pressure	Midei et al. (77)
*Postnatal:* childhood maltreatment	756 from population based study	Young adult (19–20 years)		↑ BMI in those exposed to neglect during childhood	Lissau and Sorensen (78)
				Odds ratio 9.8 CI 1.35–28.2	
*Postnatal*: childhood maltreatment	9310 of 1958 British birth cohort	45 years		↑ BMI	Thomas et al. (79)
				↑ HbA1C ≥6	
				↑ Central obesity	

Despite increasingly available epidemiological evidence, the mechanisms driving stress effects to lower birth weight remain largely unknown. Changes in HPA axis function in individuals of low birth weight have been identified. A group of adult men born with low birth weight had increased HPA axis responsiveness, which was shown to be associated with metabolic risk factors including blood pressure and increased triglycerides (80). Thus epidemiological evidence generally suggests early-life stress induces perturbed HPA axis function and alters neuroendocrine axis responsiveness. The increasing evidence demonstrating the risk of early-life stress and later metabolic disorders emphasizes the need to explore the mechanisms underlying this association. Targets for intervention, whether through pharmacological means or through lifestyle modification, need to be identified to reduce this identified risk. It is important to note that not all individuals exposed to adverse early environments develop metabolic deficits later in life; these differences may relate to genetic makeup and the environment to which the individual is exposed throughout life.

## The Effect of Later Environment on Health Outcomes Following Early-Life Stress

Early-life stress in combination with a sub-optimal later environment, such as a sedentary lifestyle, increased consumption of high energy food or persistent adulthood stress may alter the risk for developing metabolic disorders throughout life. Humans and rodents are able to habituate and adapt to the environmental conditions to which they are exposed (81–83). This adaptation occurs in prediction of exposure to similar situations in the future, and under normal conditions is of significant value, improving future resilience and coping in these situations (see Figure 2). However, it is suggested that if adaptation is inadequate, maladaptive, or future environment differs from the programed phenotype, there is increased disease susceptibility (84–87). Determining the long term consequences of early-life stress-induced changes in neuronal structure, and hormonal and nutritional status across different environments is an important public health concern. Although prevention or mitigation of early-life stress is the ideal, if through modulating the later environment (e.g., providing a positive environment) disease risk can be attenuated, important targets for intervention can be identified.

Stress throughout adulthood negatively influences lifestyle choices that are risk factors for metabolic disease, such as alterations in eating behavior, intake of high-fat food (88), drug addiction (89, 90), and reduced physical activity levels (91). Given this, it is critical to determine whether early-life stress can have a lasting influence on adult behavioral choices. Indeed, parental care factors play a key role in developing health behaviors and outcomes in children (92, 93). Parental behaviors are often imitated by children, thus a push to improve attitudes in parents, whether through reducing stress, improving eating attitudes or increasing physical activity may foster improved health status in successive generations. Recent research showed a direct association between activity levels of parents and their preschool-aged children (94). Additionally, the behavioral profile that results from early-life stress heightens the risk for impaired psychosocial function and psychiatric disorders, and this may independently influence metabolic disease risk. Future human studies must focus on these lifestyle factors during critical exposure periods and throughout adult life.

## Prenatal Stress and Impact of Later Environment: Human Studies

Prenatal exposure to adverse environments such as famine have been associated with poorer lifestyle choices including smoking incidence (66), HFD consumption, and reduced physical activity (95, 96). As explored, a possible consequence of gestational stress is preterm birth or low birth weight. Total physical activity levels (97) and non-conditioning leisure time physical activity levels (98) were not influenced by low birth weight. Despite this, adults born preterm with very low birth weight (i.e., less than 1500 g) had reduced smoking rates, yet were less likely to engage in leisure time physical activity with reduced energy expenditure than normal gestational birth controls (98). Further, healthy children aged 5–8 years old who were born prematurely were shown to have reduced physical ability to normal gestational age controls (99). Physical activity has been shown to improve health outcomes following premature birth, with 4 weeks of passive range of motion and compression exercises in premature infants shown to increase bone mineral density. The authors suggested that early exercise programs may improve physical fitness in later life (100). The unequivocal benefits of physical activity should be considered as a therapeutic tool, with activity levels inversely associated with metabolic syndrome (101). In low birth weight individuals, physical activity has been shown to modestly attenuate the association between low birth weight and insulin resistance, as assessed by HOMA-IR (102). Although this finding has not been replicated by other studies (97, 103), physical activity was identified as a better predictor of HOMA-IR comparative to birth weight a possible target for intervention (103).

## Postnatal Stress and Impact of Later Environment: Human Studies

There are limited data exploring the metabolic outcomes of postnatal stress and environment interaction. Early-life stress has been associated with later life addiction problems, including drug and food addiction (104). Adult women who were abused as children were significantly heavier and had a marked increase in food addiction risk (105). Early-life maternal stress has been positively correlated with the risk of the infant being overweight in an environment of high food security (see Table 1). Infants exposed to maternal stress during periods of food insecurity showed no significant overweight risk (74) (see Figure 2). One limitation of this study is the low socioeconomic status of the participants, as this is an individual risk factor for the development of childhood and adult obesity, amongst other health issues. Similar to these findings, the number of parental stressors and degree of perceived stress were positively associated with child obesity and increased fast-food consumption (106). Children and adolescents are susceptible to stress, and given the association between perceived stress and increased food consumption frequency and palatability (107–109), environmental factors such as palatable food access and physical activity may play a mediating factor between human early-life stress and poor metabolic outcomes.

Physical activity may facilitate a protective role against childhood stressors, with more active children showing reduced salivary cortisol response to stressful situations, reflective of lower HPA axis reactivity (110). In addition to alterations in stress mediators, childhood maltreatment has been independently associated with elevated levels of inflammatory markers in adulthood (76, 111) (see Table 1). Increased inflammation following early-life stress is clinically relevant and may provide an important causal link between adverse early-life experience and adulthood metabolic risk. The benefits of physical activity on immune function and inflammation are well-established, with reductions in inflammatory biomarkers associated with disease (112).

## Prenatal Stress and Impact of Later Environment: Animal Studies

Given the global epidemic of metabolic disease, examining the growing body of animal evidence linking prenatal stress with impaired metabolic profile when exposed to energy dense, palatable diets is imperative. The effects of stress during pregnancy on metabolic consequences in later life are well characterized relative to stress during the early postnatal period (22). Identifying how programed changes during early life adversely impact susceptibility to later life nutrition is critical and could provide targets for intervention (see Figure 2). Two common models of prenatal stress exposure involve subjecting dams to a single stressor during gestation, for instance restraint stress. Alternatively, dams can be exposed to a combination of variable stressors such as restraint, air puff startle, forced swimming, starvation, and bright light exposure (113).

Evidence to support a programed resilience to later life metabolic deficits following prenatal early-life stress has been observed in some animal studies. For example, independent of body weight, adult female offspring of stressed dams consuming standard laboratory chow, had a lower insulin area under the curve (AUC) during an oral glucose tolerance test (OGTT) (114), reduced plasma insulin, and improvements in HOMA-IR (24) compared to offspring of non-stressed dams. This improvement in insulin sensitivity is suggestive of a prenatal early-life stress programing of glucose handling in insulin sensitive tissues. However, it is unknown if these improvements in insulin sensitivity following prenatal early-life stress will be sustained as animals age. Exposure of offspring to a later negative environment following prenatal stress, such as an HFD, induces maladaptation, rather than resilience (see Figure 2). Offspring of stressed dams weaned onto an HFD showed alterations in metabolic parameters with female rats shown to have an elevated glucose AUC during an OGTT, relative to control and stress-chow groups, whilst HFD fed males of stressed dams required a greater amount of insulin to clear a given glucose load relative to control (114). In a similar study, prenatal stress did not alter glucose clearance as assessed through intraperitoneal glucose tolerance test (IPGTT), yet, female offspring exposed to stress had increased visceral and retroperitoneal fat depot mass after 10 weeks on a high-fat sucrose diet relative to unstressed controls consuming the same diet (24).

The mechanisms by which prenatal stress alters later life metabolic outcome remain unknown. Stress during pregnancy alters maternal hormones, including circulating glucocorticoid levels. Normal physiological levels of glucocorticoids during development are essential for tissue growth and maturation, however, excess levels of glucocorticoids, e.g., through pharmacological interventions, have been shown to affect maturation (115, 116). Elevated maternal glucocorticoid can cross the placenta (117), and this has been shown to affect growth, morphology, and function of brain and peripheral tissues during fetal development (118). Seckl (119) has demonstrated a stress-mediated mechanism that underpins the low birth weight and increased risk for adulthood health deficits; exposure to increased level of glucocorticoids either synthetically (dexamethasone) during late gestation or via stress (malnutrition, adverse environment exposures) reduces birth weight and impacts maturation of major organs (119, 120). Dams exposed to prenatal variable stress were shown to have heavier adrenal mass and lower fecal corticosterone secretion vs. non-stressed controls (121). This is suggestive of a reduced corticosterone clearance in these stressed dams during gestation. Excess glucocorticoid exposure during pregnancy has also been shown to affect glucose and insulin metabolism (17, 122, 123).

To model periods of nutritional stress in humans, such as famine, reduced nutritional availability during gestation can be used, enabling investigation of the developmental and programing outcomes on offspring (124–126). Restricting dams to 30% of normal food intake, led to low birth weight offspring; pups who were undernourished then weaned onto HFD were found to have significantly elevated leptin, C-peptide, insulin, and body fat compared to control-HFD pups. Notably, injection with leptin for 10 days during lactation (PND3–13) completely normalized these markers (127), suggesting normal maternal levels of leptin are important during development. Food intake and activity levels are also influenced by nutritional insult during gestation. Offspring of female Wistar rats undernourished during gestation (30% of *ad libitum*) showed significantly reduced locomotor activity with marked hyperphagia and hyperleptinemia when consuming either standard chow or a high caloric diet (128). Leptin is a critical adipocyte derived hormone known to play an essential role in the regulation of feeding and in the maintenance of energy homeostasis. Adult rats exposed to a single 24-h deprivation period during the lactation period demonstrated marked reductions in leptin, although this is difficult to interpret due to the nutritional insult that would have accompanied 24 h of starvation (40, 129, 130).

## Postnatal Stress and Impact of Later Environment: Animal Studies

Various models of postnatal early-life stress have explored the influence of later life environmental insults on metabolic function, each of which seem to elicit different outcomes and sex-specific effects (see Table 2). Work in our lab demonstrated HFD fed male rats previously exposed to maternal separation, have marked elevations in plasma insulin, and decreased total white adipose tissue mass, independent of body weight vs. HFD controls (131, 132). In agreement, maternal deprivation induced early adulthood hyperinsulinemia and impairments in insulin sensitivity, measured through HOMA-IR, in male offspring fed with an HFD relative to HFD controls (130). Similar metabolic changes are observed in maternal deprivation exposed female offspring, with HFD shown to cause early adulthood hyperinsulinemia, at PND35 compared to PND102 in control rats consuming an HFD (130). Further, maternally deprived female rats consuming a high-fat sucrose diet had a trend for decreased fat depot mass vs. control (121). Less work has examined the metabolic profile arising subsequent to early-life stress induced by LN material. A recent study explored for the first time the metabolic profile in Wistar female rats; showing reduced body weight at weaning, and reduced food intake, suggesting altered energy utilization and storage. Interestingly, these pups had exaggerated HPA axis activity with delayed clearance of corticosterone from the circulation, and taken together these data further suggest an early-life stress-induced interaction between the HPA axis and metabolic profile (133) (see Table 2).

**Table 2:** **Postnatal early-life stress and metabolic consequences in rodents**.

Offspring	Stress protocol	Other interventions	Metabolic consequences	Reference
Male Wistar rats	*MS*: separation for 240 min daily from PND1 to 10	*Diet*: PND21–35: standard chow	*MS vs. control* ↑ Food intake and bodyweight at weaning ↑ Gonadal and retroperitoneal WAT ↑ Plasma triglycerides *MS-deficient diet vs. control-adequate and control-deficient*	Bernardi et al. (134)
	*Control*: non-handled	PND35 to cull: *n*-3 polyunsaturated fatty acid adequate or deficient diet		
			↑ Plasma leptin	
			*MS-deficient vs. MS-adequate and control-deficient*	
			↑ Fasting plasma insulin	
			↑ HOMA-IR index	
Male Sprague-Dawley rats	*MS*: separation for 180 min daily from PND1 to 14	*Social isolation*: weaned into group housing (*n* = 3 per cage) or isolation (single rat)	*MS vs. control* ↑ Bodyweight at weaning	Ryu et al. (135)
	*Control*: non-handled			
			*MS-isolation vs. MS-group*	
			↑ Bodyweight from PND42	
			↑ Food intake at PND42 and 56	
			*Control-group vs. control-isolation*	
			No significant effect on weight gain with isolation	
Female Sprague-Dawley rats	*MS*: separation for 180 min daily from PND10 to 15	*Diet*: high-fat diet (HFD)	No change in bodyweight	Miki et al. (136)
			*Retroperitoneal WAT at 10 weeks of age*	
			↑ Prohibitin mRNA in MS rats compared to control (*P* < 0.001)	
	*Control*: non-handled		*Interscapular BAT at 10 weeks of age*	
			↓ β3-Adrenergic receptor mRNA in MS rats compared to control (*P* < 0.001)	
			No change in UCP-1 mRNA across groups	
Male and female Sprague-Dawley rats	*MS*: separation for 180 min daily from PND2 to 14	*Diet*: weaning to cull: standard chow or cafeteria style HFD	*MS-chow vs. control* ↑ Plasma corticosterone following restraint stress	Maniam and Morris (131, 132)
	*Control*: 15 min daily from PND2 to 14	*Exercise*: weaning to cull: exercise (voluntary running wheels) or sedentary (locked running wheels)		
			↓ Hippocampal GR mRNA expression	
			Reversed with HFD or exercise	
			*MS-HFD vs. control-HFD*	
			↑ Plasma insulin	
			↓ Total WAT per gram bodyweight	
			*MS-chow-exercise vs. MS-chow-sedentary*	
			↓ Plasma corticosterone following restraint stress	
			*MS-HFD-exercise vs. MS-HFD-sedentary*	
			↓ Plasma insulin	
Male and female Wistar rats	*MD*: 24 h maternal deprivation from PND9 to 10	*Diet*: weaning to cull: standard chow or HFD	*MD-chow vs. control* ↓ Plasma leptin	Mela et al. (130)
	*Control*: non-handled			
			Reversed by HFD consumption	
			*MD-HFD vs. MD-chow and control-HFD*	
			↑ Hypothalamic IL-1β and TNF-α mRNA	
			*MD-HFD males vs. MD-chow and control males*	
			↑ HOMA-IR	
Male and female Wistar rats	*MD*: 24 h maternal deprivation from PND9 to 10	*Diet*: standard chow	*MD vs. control*	Viveros et al. (40)
			↓ Bodyweight until 40–50 days of age	
			↓ Plasma leptin at PND75	
			*MD males vs. control males*	
	*Control*: non-handled		↓ Plasma testosterone	
			↓ PPAR-α mRNA in perirenal adipose tissue at PND35	
			*MD females vs. control females*	
			↓ Plasma adiponectin at PND75	
Male and female Sprague-Dawley rats	*LN*: dams and pups subject to limited nesting material from PND2 to 9	*Diet*: standard chow	*LN vs. control at PND9* ↓ Bodyweight	Avishai-Eliner et al. (137)
	*Control*: normal bedding			
			↑ Plasma corticosterone and adrenal weight	
			↓ CRH mRNA in hypothalamic paraventricular nucleus	
			↓ GR mRNA in hypothalamic paraventricular nucleus and frontal cortex	
Female Wistar rats	*LN*: dams and pups subject to limited nesting material from PND2 to 9 *Control*: normal bedding	*Diet*: weaning to PND111: standard chow PND111–141: standard chow or chow plus HFD	*LN vs. control* ↓ Bodyweight ↑ Consumption of palatable HFD as a percentage of total food intake	Machado et al. (133)
		Following which rats underwent a 24-h food preference test		
			Prior chronic exposure to HFD did not decrease preference for palatable food in LN rats, whereas control demonstrated reduced preference for HFD	
Male and female C56BL/6J mice	*LN*: dams and pups subject to limited nesting material from PND2 to 9	*Diet*: standard chow	*LN vs. control at PND9* ↓ Bodyweight, positively correlated to amount of nesting material	Rice et al. (45)
	*Control*: normal bedding			
			↑ Plasma corticosterone	
			↓ CRH mRNA in hypothalamic paraventricular nucleus	
			*LN vs. control at adulthood*	
			Restored bodyweight	
			↑ Plasma corticosterone	
			↓ CRH mRNA in hypothalamic paraventricular nucleus	

Few studies have explored biological characteristics of peripheral tissues following early-life stress. Recently, maternally deprived female rats were shown to have significantly reduced brown adipose tissue β3-adrenergic receptor mRNA expression and increased white adipose tissue prohibitin mRNA relative to control, with no change in UCP-1 (136). The authors concluded these results may facilitate adipose tissue proliferation later in life. An altered response to nutritional challenge following early-life stress is not only observed in high caloric fed states. Maternally separated rats consuming an omega-3 deficient diet demonstrated marked elevations in plasma insulin and impaired insulin sensitivity, as assessed by HOMA-IR, relative to control animals consuming the same diet (134). Functional studies performed by our lab have demonstrated attenuated adulthood outcomes of early-life stress when siblings are provided with a positive environment of voluntary running wheel exercise (see Figure 2). Male rats exposed to maternal separation and weaned onto a standard chow diet demonstrated a hyper-responsive corticosterone response to novel restraint stress, which was dampened with exercise and HFD. Impaired changes in metabolic parameters of insulin and diet-induced obesity were also attenuated with exercise, reversing maternal separation-induced hyperinsulinemia and increased body weight relative to HFD controls and stressed sedentary counterparts (132).

A common aspect across early-life stress models centers around glucocorticoid exposure and HPA axis activation during the stress-hypo-responsive period, a stage of neonatal resilience to mild stressors suggested to likely trigger corticosterone secretion in adult life (84). Synthetic glucocorticoid administration attempts to replicate stress models, as this elicits an HPA axis response during the early postnatal period in rodents (41, 138). This also allows for controlled dose administration and with necessary caution can improve understanding of the impact of glucocorticoid exposure during these critical periods. Early-life corticosterone administration reduced adult female rat body weight and decreased fat depot mass relative to control (139). Studies of glucocorticoid administration highlight the marked influence of combining stress hormones and additional metabolic insults. In young male Sprague-Dawley rats, glucocorticoid administration alone did not alter insulin sensitivity, assessed by HOMA-IR, or glucose disposal following an OGTT (140). Conversely, consumption of a palatable HFD in combination with glucocorticoid exposure is known to induce a marked increase in fasting plasma glucose and insulin, with impaired glucose clearance following OGTT (140, 141). Further investigation is required to determine whether the unaltered or improved insulin sensitivity with chow consumption would prove deleterious in the long term (as shown in Figure 2).

## Early-Life Stress and Programing of Peripheral Tissues

### Mechanisms underlying early-life stress-induced metabolic deficits: Role of the HPA axis, glucocorticoid, and tissue 11β-HSD1

To uncover the mechanisms underlying early-life stress-induced metabolic derangements, it is first essential to understand the action of glucocorticoids at different concentrations on glucose and insulin homeostasis and lipid metabolism. At pharmacological doses, glucocorticoids act as potent anti-inflammatory agents but high levels of circulating glucocorticoids result in metabolic derangements including increased visceral adiposity, dyslipidemia (increased levels of triglycerides), increased non-esterified fatty acids (NEFA) (142–144), and impaired glucose and insulin tolerance (145–147). In contrast to the anabolic actions of insulin, glucocorticoids are predominantly catabolic, decreasing glucose utilization and insulin sensitivity with both human and animal data revealing insulin intolerance with excess exposure (145, 148).

**Figure 1:**
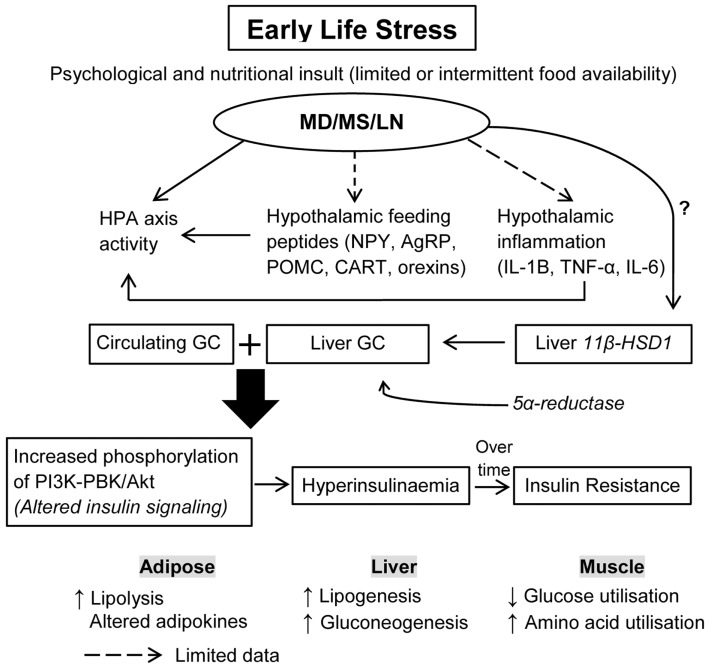
**How does ELS increase the risk for insulin resistance and hyperglycemia?** Early-life stress (ELS) induced by three different paradigms including maternal deprivation, maternal separation, and limited nesting material are known to dysregulate HPA axis activity, with limited data on the effects of ELS on hypothalamic feeding neuropeptides and inflammation. It is proposed that ELS disturbs circulating glucocorticoids (GC) through a combined action of HPA axis activity, hypothalamic feeding neuropeptides, and inflammatory changes. The effects of ELS on liver 11β-HSD1, an enzyme that converts inactive GC to active GC, and 5α-reductase, an enzyme involved in GC metabolism, is less known. It is proposed that during the maladaptation period, ELS affects tissue levels of these enzymes, thus increasing exposure of peripheral tissues to GC. Excess GC availability can alter insulin signaling, leading to hyperinsulinemia and insulin resistance over time. Thus, increases in circulating and tissue GC induced by ELS act synergistically to exacerbate insulin resistance in peripheral tissues and alter energy expenditure and utilization. ELS, early-life stress; MD, maternal deprivation; MS, maternal separation; LN, limited nesting material; GC, glucocorticoids; TNF-α, tumor necrosis factor alpha; IL-6, interleukin 6; IL-1β, interleukin-1 beta; 11β-HSD1, 11-beta hydroxysteroid dehydrogenase.

Glucocorticoids exert tissue-specific metabolic effects, directly targeting tissues for insulin metabolism, and they regulate skeletal muscle, liver, and adipocyte insulin signaling (149). Glucocorticoids alter glucose and protein metabolism; increased levels of glucocorticoids induced during stress increase protein degradation, which results in the generation of amino acids that serve as precursor for glucose synthesis in the liver. In addition, excess glucocorticoids inhibit glucose uptake into muscle by inhibiting translocation of glucose transporter-4 (150–152). Chronically increased circulating or tissue glucocorticoid levels may also lead to insulin resistance, hypertriglyceridemia, and hepatic steatosis (see Figure 1). Circulating glucocorticoid concentrations are tightly controlled by activation of the HPA axis, however, tissue-specific availability is regulated by multiple means including glucocorticoid receptor expression, receptor affinity, and alterations in glucocorticoid metabolism and clearance.

Mechanisms regulating intracellular glucocorticoid concentrations are critical to understand the impact of stress on energy metabolism including energy expenditure, storage, and utilization. Intracellular levels of glucocorticoids are influenced by 11β-HSD1 with the type-1 isoform predominantly expressed in the liver (153) and to a lesser degree in adipose and skeletal muscle (154). Evidence shows that tissue glucocorticoid levels are regulated by 11-beta hydroxysteroid dehydrogenase (11β-HSD1) in target tissues (155) as 11β-HSD1 converts inactive cortisone to biologically active cortisol (156, 157). The liver is a major site of glucocorticoid metabolism where 11β-HSD1 regulates the access of glucocorticoid to the glucocorticoid receptor, leading to glucocorticoid metabolism which, is regulated by 5-alpha/beta reductase levels (155, 158). Fat, liver, and muscle-specific increases in 11β-HSD1 are known to increase the risk for metabolic disorders such as insulin resistance, hyperglycemia, and hyperlipidemia (159, 160). Tissue glucocorticoid amplifies the action of insulin to promote lipogenesis within hepatocytes (161). 11β-HSD1 in the liver increases glucocorticoid action in liver to stimulate gluconeogenesis and inhibit beta-oxidation of fat, thus promoting lipid synthesis (162–164). A very recent study demonstrated reduced 5-alpha reductase was associated with fatty liver (165). Thus liver-specific glucocorticoid synthesis and clearance regulated by 11β-HSD1 and 5-alpha reductase appear to affect hepatic lipid accumulation. Interestingly, animal studies demonstrate a link between hepatic glucocorticoid metabolism with regulation of HPA axis activity and lipid synthesis in the liver. For example, transgenic overexpression of 11β-HSD1 in liver of null mice normalized the exaggerated HPA axis activity in response to stress, and led to fatty liver (166, 167). On the other hand, hepatic deletion of 11β-HSD1 led to hyperactivity of the HPA axis (168). These studies suggest liver 11β-HSD1 greatly contributes to amplify circulating glucocorticoid levels, and thus likely mediates the negative feedback activity to dampen HPA axis activity, a concept previously proposed by Chapman et al. (169). Several studies have demonstrated a blunted activity of the HPA axis in response to novel stress following high energy diets such as high sugar or HFD either after adulthood chronic stress or chronic stress exposure during early life (131, 132, 170). As these studies did not report measures of liver 11β-HSD1, it is not clear whether under-activity of the HPA axis following early-life stress and postnatal high energy diet is modulated by liver 11β-HSD1 levels.

Thus, liver 11β-HSD1-HPA axis is a potential pathway in early-life stress-mediated metabolic disturbances, particularly insulin sensitivity, glucose metabolism and lipid synthesis, and mobilization as outlined in Box 1. This hypothesis, however, needs systematic examination in the future. Early-life stress, modeled through prenatal dexamethasone treatment, has been shown to upregulate 11β-HSD1 in peripheral tissues such as liver, pancreas, and subcutaneous fat in rat offspring at 4 months with persistent increases at 1 year of age (171). Interestingly, it was previously shown that early-life stress alters the expression of liver 5-alpha reductase mRNA (172, 173). Thus, this suggests a programing effect of early-life stress on tissue 11β-HSD1 expression, glucocorticoid metabolism, and glucocorticoid signaling. There has been limited evidence regarding early postnatal stress effects on 11β-HSD1 expression and glucocorticoid metabolism of peripheral tissues, which needs to be explored in future studies (Figure 1).

Box 1 **Outstanding research questions/proposals.**

1. Does chronic exposure to negative postnatal environments such as HFD and adulthood stressors lead to maladaptation following early-life stress?
2. Is the stress-induced perturbation of metabolic profile mediated by the liver 11β-HSD1-HPA axis interaction?
3. How can positive postnatal environments reverse the early-life stress-induced metabolic damage?
4. What are the mechanism(s) underlying early-life stress-induced improvement of insulin sensitivity and improved glucose metabolism if rodents are maintained on chow diet?

**Figure 2:**
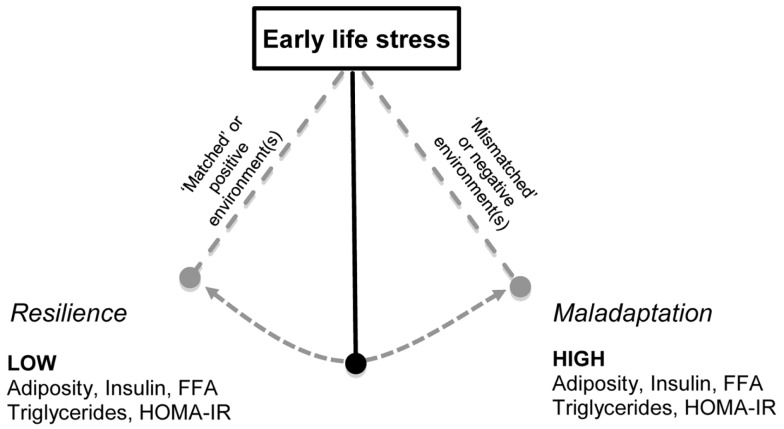
**The combination of early-life stress exposure with altered later environment may determine metabolic outcomes**. Early-life stress (ELS) exposure during gestation or the postnatal period is hypothesized to influence an offspring’s response to later environments (85, 87). This programing occurs in an attempt to facilitate habituation and resilience to future similar situations. Offspring exposed to environments that do not differ to that to which they were exposed during early life, i.e., “matched” or positive environments, such as exposure to exercise have been shown to adapt and demonstrate resilience (74, 114, 131, 132). Conversely, exposure to a negative environment, i.e., “mismatched,” such as a sub-optimal diet (131, 132, 134) or chronic stress following ELS may lead to maladaptation, and metabolic deficits, with increased levels of triglycerides, free fatty acids, adiposity, and insulin resistance as measured by HOMA-IR. Thus, there is a pendulum of vulnerability and the trajectory following ELS is influenced by the later life environment.

We propose that early-life stress may alter availability of tissue glucocorticoids and glucocorticoid signaling. Specifically, we propose that during the maladaptation period (see Figure 2), early-life stress enhances availability of tissue glucocorticoids via increases in liver 11β-HSD1. The tissue glucocorticoid-induced insulin resistance may involve glucocorticoids altering insulin signaling via increasing phosphorylation of PKB/akt, which stimulates insulin secretion, that is, glucocorticoids work synergistically with insulin which increases adipocyte lipolysis and liver lipogenesis (see Figure 1). Another possibility is that early-life stress impairs glucocorticoid signaling involving glucocorticoid–glucocorticoid receptor binding and phosphorylation of the glucocorticoid receptor. Impairment in glucocorticoid signaling leads to alteration in glucocorticoid targeted genes that regulate hepatic glucose production, and hepatic lipogenesis including peroxisome proliferator-activated receptor gamma, coactivator 1 alpha (PGC1-alpha), phosphoenolpyruvate carboxykinase (PEPCK), glucose 6-phosphate (G6P), and diacylglycerol acyltransferase (DGAT). Genes known to mediate hepatic lipid accumulation such as PGC1-alpha and adipose DGAT1 were altered in rat pups from dams that had been subjected to prenatal stress (174). PGC1-alpha plays an essential role in fatty acid oxidation while increased DGAT1 in adipose tissue increases lipogenesis. DGAT1 transgenic mice fed with an HFD demonstrated a 300% increase in liver triglycerides suggesting a redistribution of the fat from adipose tissue to liver via re-esterification of fatty acid with glycerol (175). Another study showed that prenatal stress induced through manipulation of the availability of food, that is either by limiting intake, or exposing to a high energy food during pregnancy led to fatty liver in pups relative to controls (176).

In conclusion, the role of liver 11β-HSD1 in regulating HPA axis activity, and whether it is modulated by stress early in life, warrants investigation. We propose that early-life stress induces changes in glucocorticoid metabolism and signaling, likely mediating the metabolic consequences reported. The question of how these affect glucocorticoid-induced insulin dependent processes, including hyperinsulinemia and lipid metabolism following an early-life stress exposure will be addressed in the following section.

## Proposal of How ELS may Increase Risk of Insulin Resistance, and Hyperglycemia

It has been known for decades that stress early in life in both humans and animals can affect HPA axis activity in later life (131, 132, 177–179), however, the effects of early-life stress on hypothalamic neuropeptides involved in feeding are less well known, with only a few studies exploring hypothalamic neuropeptides following maternal separation or deprivation (131, 180). Feeding regulation, which is tightly regulated by orexigenic and anorexigenic hypothalamic neuropeptides, is also influenced by HPA axis activity and circulating glucocorticoid concentrations (181, 182). Glucocorticoids stimulate feeding responses by increasing the release of neuropeptide Y (NPY) and inhibiting that of corticotrophin releasing hormone in the hypothalamus however the orexigenic effect of glucocorticoids may be counteracted by leptin (183).

Mela and colleagues explored the effect of 24 h of maternal deprivation on postnatal day 9 in rats on hypothalamic feeding neuropeptides measured at 14 weeks of age in rats fed with chow or HFD (130). No significant differences in orexigenic (NPY, agouti-related peptide (AgRP) and anorexigenic (pro-opiomelanocortin and cocaine- and amphetamine-regulated transcript) neuropeptides were observed between the chow and HFD fed rats. An interesting finding in this study was that while HFD did not alter these neuropeptides in control females, rats that experienced maternal deprivation had significantly decreased hypothalamic NPY and AgRP mRNA expression and a significant increase in hypothalamic pro-opiomelanocortin and cocaine- and amphetamine-regulated transcript relative to their chow counterparts. The increased caloric intake by HFD fed rats relative to their chow counterparts was similar between the maternally deprived and control rats. But an increased plasma leptin concentration in the maternally deprived HFD fed female rats suggests a programing effect of maternal deprivation which dysregulated feeding homeostasis. Whether this may be linked to either the HPA axis activity or tissue-specific availability of glucocorticoids needs to be explored in the future. The significantly reduced fasting triglycerides and lack of change in fasting insulin by HFD feeding is puzzling, and these may be related to either the strain or the diet used. Thus, use of appropriate controls and dietary conditions are critical to enable exploration of the underlying mechanisms linking combined early-life stress and high-fat feeding with later metabolic risk. Overall, limited studies have directly investigated whether early-life stress affects hypothalamic neuropeptides and inflammation.

The increased availability of glucocorticoids discussed above may play a role in altering tissue insulin signaling by increasing the phosphorylation of PKB/akt. Animals exposed to early-life stress may be resilient to the increased availability of tissue glucocorticoids and thus subsequently show a dampened secretion of insulin. While there are no systematic studies on early-life stress and metabolic consequences, there is evidence in rodents showing early-life stress does not affect basal metabolic hormones if animals consume chow, including non-fasted plasma insulin and leptin (131, 132). Another study demonstrated improved insulin sensitivity following early-life stress when measured during adulthood (170). Taken together, we propose that early-life stress may lead to resilience, and thus the organism potentially may adapt to any changes in the postnatal environment, such as exposure to high energy diet, or later stressors through enhanced negative feedback sensitivity of the HPA axis activity and reprograming of the peripheral tissue sensitivity to glucocorticoid exposure (see Figure 2). However, if an organism exposed to early-life stress is chronically exposed to these negative postnatal environments (high energy diet and later stressors), the enhanced negative feedback sensitivity maybe dysregulated, resulting in perturbed HPA axis activity, leading to a phase known as maladaptation (see Figure 2). Indeed there is evidence that such maladaptations can influence glucose/insulin homeostasis, resulting in the manifestation of metabolic disorders including insulin resistance, hyperglycemia, hyperlipidemia, as suggested in Figure 2.

During periods of maladaptation, increased tissue glucocorticoids and circulating corticosterone will impair the insulin signaling pathway, leading to hypersecretion of insulin; a condition leading to insulin resistance (see Figure 2). This will mediate tissue specific effects, that is, increased adipose lipolysis through beta-oxidation which releases free fatty acids into the circulation and re-esterification in the liver to promote lipogenesis. In addition, increased tissue glucocorticoid levels via insulin stimulation increases hepatic glucose production. Prenatal stress has been shown to increase hepatic PEPCK mRNA and this was enhanced with high energy feeding (174). There are no data thus far on the impact of early postnatal stress on hepatic glucose production, which is an important measure to be considered for future studies to improve understanding of the link between postnatal stress and risk of pre- or diabetes. In addition, increased glucocorticoid-induced hyperinsulinemia also alters the muscle glucose utilization via affecting the glucose transporter gene and expression, including the GLUT4-transporter.

## Conclusion and Future Directions

Despite significant progress in the field of early life programing and metabolic disease risk many challenges exist. Human studies are vital in providing evidence of the association between early-life adversity and disease incidence but data must be interpreted with necessary caution. A majority of human evidence is based on parental or offspring self-report raising the possibility of confounding due to issues with information recall, lack of accuracy, and the potential for bias.

Future studies in humans should seek to better quantify stress exposure during the early-life period and at the time of assessment to improve knowledge of how different stress types may alter disease risk. Given both environment and genetic predisposition determine health outcome, studies should not only consider stressor experience during early life but also control for socioeconomic status, food and education availability, ethnicity, and lifestyle factors such as nutritional status, smoking, and physical activity throughout their lifetime.

According to the hypothesis explored throughout this review, programed adaptation during early life occurs in an attempt to adapt to the predicted later life environment and hence even seemingly trivial variations in stressors during these periods may vary the observed outcomes. Inconsistency in findings across experiments could be due to the marked differences in study design. Procedural variations in maternal separation have been reviewed elsewhere (177). In the literature, there is no consistent procedure, rather multiple experimental conditions fall under the broad term of maternal separation. Thus, duration, and age at separation, temperature in which pups are separated, whether pups are isolated from litter mates during separation and whether pups are removed or remain in their home cage have all been shown to influence behavioral outcomes and brain function. Animal models must consider the influence of maternal care on long term outcomes, which is a major aspect the novel model of limiting nesting material attempts to explore. Work exploring prenatal or gestational stress exposure could benefit from cross-fostering to better delineate effects of maternal stress and the influence of received postnatal care. Offspring of low care dams that were cross-fostered by high-licking high arched back nursing dams were resilient in terms of the decline in hippocampal synaptogenesis and spatial learning seen in offspring reared by low care dams (184).

Animal research should ultimately aim to improve public health outcomes. To ensure this, analysis of behavioral, physiological, and molecular parameters is required. The current literature lacks assessment of whole body insulin sensitivity measures or assessment of β-cell structural changes – key factors that influence metabolic outcome. Models need to reflect the human condition upon which they are based, which brings the need for valid controls. Given the complex heterogeneity of both the stress system and metabolic disease the phenotype of experimental animals would ideally be comprehensively assessed, rather than examining single factors. Species and strain differences must be considered and these animals must be valid models for the environmental conditions that the study aims to investigate.

Investigation of the environmental influence provides us with an opportunity to better identify factors determining vulnerability and resilience to early-life experience. A better understanding of factors driving the association between genetic predisposition with both early and adult environment will help with the identification of targets for intervention with the hope of minimizing disease incidence. As known, high energy feeding impairs glucose and insulin tolerance and affects lipid metabolism, thus chronic HFD could serve as a good model to study the altered effects of early-life stress on metabolism. Our lab and others have shown early evidence that HFD or altered nutrition lead to altered insulin levels in early-life stressed animals relative to control (131, 132, 134, 170). Together these studies appear to suggest that both short term diet and long term diet exaggerate plasma insulin. Despite this, a single marker or circulating hormone levels limit ability to draw conclusions of how early-life stress may exert negative impact on insulin sensitivity and overall metabolic risk. Future studies need to adopt a mechanistic approach, examining animals using appropriate metabolic tests that will provide answers to the outstanding questions outlined in Box 1. An alternate model of early-life stress, variable foraging demand, demonstrated impaired insulin resistance in non-human primates as measured by hyperglycemic-insulin clamp (185). Functional studies in rodent early-life stress models, such as glucose or insulin tolerance tests, or preferably glucose clamp, would be useful to explore this further.

In conclusion, the mechanisms whereby adverse early-life events accelerate metabolic deficits have received little attention to date. When combined with a sub-optimal subsequent environment (e.g., poor diet, stress, physical inactivity) early-life stress may exacerbate the risk of metabolic disease. One potential mechanism underlying early-life stress-induced metabolic deficits is the interaction between the HPA axis and liver 11β-HSD1. Positive later environments may modulate the negative impact of early-life stress not only on behavioral outcomes, but also on metabolism. Given the burgeoning issues of metabolic and mental health disorders, the question of how early-life stress impacts subsequent disease risk warrants further investigation.

## Conflict of Interest Statement

The authors declare that the research was conducted in the absence of any commercial or financial relationships that could be construed as a potential conflict of interest.

## References

[1] Chrousos GP Stress and disorders of the stress system. Nat Rev Endocrinol (2009) 5:374–81 10.1038/nrendo.2009.106 19488073

[2] De Kloet ER Derijk R Signaling pathways in brain involved in predisposition and pathogenesis of stress-related disease: genetic and kinetic factors affecting the MR/GR balance. Ann N Y Acad Sci (2004) 1032:14–34 10.1196/annals.1314.003 15677393

[3] McEwen BS Gianaros PJ Central role of the brain in stress and adaptation: links to socioeconomic status, health, and disease. Ann N Y Acad Sci (2010) 1186:190–222 10.1111/j.1749-6632.2009.05331.x 20201874 PMC2864527

[4] Dallman MF Akana SF Laugero KD Gomez F Manalo S Bell ME A spoonful of sugar: feedback signals of energy stores and corticosterone regulate responses to chronic stress. Physiol Behav (2003) 79:3–12 10.1016/S0031-9384(03)00100-8 12818705

[5] Dallman MF La Fleur SE Pecoraro NC Gomez F Houshyar H Akana SF Minireview: glucocorticoids – food intake, abdominal obesity, and wealthy nations in 2004. Endocrinology (2004) 145:2633–8 10.1210/en.2004-0037 15044359

[6] Dallman MF Akana SF Pecoraro NC Warne JP La Fleur SE Foster MT Glucocorticoids, the etiology of obesity and the metabolic syndrome. Curr Alzheimer Res (2007) 4:199–204 10.2174/156720507780362236 17430247

[7] Oda N Nakai A Mokuno T Sawai Y Nishida Y Mano T Dexamethasone-induced changes in glucose transporter 4 in rat heart muscle, skeletal muscle and adipocytes. Eur J Endocrinol (1995) 133:121–6 10.1530/eje.0.1330121 7627333

[8] McEwen BS Steroid hormones: effect on brain development and function. Horm Res (1992) 37(Suppl 3):1–10 10.1159/000182393 1330863

[9] Munck A Koritz SB Studies on the mode of action of glucocorticoids in rats. I. Early effects of cortisol on blood glucose and on glucose entry into muscle, liver and adipose tissue. Biochim Biophys Acta (1962) 57:310–7 10.1016/0006-3002(62)91124-1 14477182

[10] Issekutz B Jr Shaw WA Glucose turnover in the exercising dog with chemically induced diabetes and the effect of methylprednisolone. Diabetes (1975) 24:915–21 10.2337/diab.24.10.915 126184

[11] Calabrese F Molteni R Racagni G Riva MA Neuronal plasticity: a link between stress and mood disorders. Psychoneuroendocrinology (2009) 34(Suppl 1):S208–16 10.1016/j.psyneuen.2009.05.014 19541429

[12] Pruessner JC Dedovic K Pruessner M Lord C Buss C Collins L Stress regulation in the central nervous system: evidence from structural and functional neuroimaging studies in human populations – 2008 Curt Richter Award Winner. Psychoneuroendocrinology (2010) 35:179–91 10.1016/j.psyneuen.2009.02.016 19362426

[13] Heim C Binder EB Current research trends in early life stress and depression: review of human studies on sensitive periods, gene-environment interactions, and epigenetics. Exp Neurol (2012) 233:102–11 10.1016/j.expneurol.2011.10.032 22101006

[14] Bjorntorp P Holm G Rosmond R Hypothalamic arousal, insulin resistance and type 2 diabetes mellitus. Diabet Med (1999) 16:373–83 10.1046/j.1464-5491.1999.00067.x 10342336

[15] Bjorntorp P Rosmond R Hypothalamic origin of the metabolic syndrome X. Ann N Y Acad Sci (1999) 892:297–307 10.1111/j.1749-6632.1999.tb07803.x 10842670

[16] Buchmann AF Kopf D Westphal S Lederbogen F Banaschewski T Esser G Impact of early parental child-rearing behavior on young adults’ cardiometabolic risk profile: a prospective study. Psychosom Med (2010) 72:156–62 10.1097/PSY.0b013e3181c88343 19995883

[17] Lesage J Del-Favero F Leonhardt M Louvart H Maccari S Vieau D Prenatal stress induces intrauterine growth restriction and programmes glucose intolerance and feeding behaviour disturbances in the aged rat. J Endocrinol (2004) 181:291–6 10.1677/joe.0.1810291 15128277

[18] Nemeroff CB Neurobiological consequences of childhood trauma. J Clin Psychiatry (2004) 65(Suppl 1):18–28 14728093

[19] Cohen RA Grieve S Hoth KF Paul RH Sweet L Tate D Early life stress and morphometry of the adult anterior cingulate cortex and caudate nuclei. Biol Psychiatry (2006) 59:975–82 10.1016/j.biopsych.2005.12.016 16616722

[20] Foscolo DR Foscolo RB Marubayashi U Reis AM Coimbra CC Neonatal maternal separation affects endocrine and metabolic stress responses to ether exposure but not to restraint exposure in adult rats. Metab Brain Dis (2008) 23:375–85 10.1007/s11011-008-9102-9 18923888

[21] Carpenter LL Tyrka AR Ross NS Khoury L Anderson GM Price LH Effect of childhood emotional abuse and age on cortisol responsivity in adulthood. Biol Psychiatry (2009) 66:69–75 10.1016/j.biopsych.2009.02.030 19375070 PMC2696583

[22] Cottrell EC Seckl JR Prenatal stress, glucocorticoids and the programming of adult disease. Front Behav Neurosci (2009) 3:19 10.3389/neuro.08.019.2009 19826624 PMC2759372

[23] Brunton PJ Sullivan KM Kerrigan D Russell JA Seckl JR Drake AJ Sex-specific effects of prenatal stress on glucose homoeostasis and peripheral metabolism in rats. J Endocrinol (2013) 217:161–73 10.1530/JOE-12-0540 23428582

[24] Paternain L de la Garza AL Batlle MA Milagro FI Martinez JA Campion J Prenatal stress increases the obesogenic effects of a high-fat-sucrose diet in adult rats in a sex-specific manner. Stress (2013) 16:220–32 10.3109/10253890.2012.707708 22738222

[25] de Vries A Holmes MC Heijnis A Seier JV Heerden J Louw J Prenatal dexamethasone exposure induces changes in nonhuman primate offspring cardiometabolic and hypothalamic-pituitary-adrenal axis function. J Clin Invest (2007) 117:1058–67 10.1172/JCI30982 17380204 PMC1821070

[26] Seckl JR Holmes MC Mechanisms of disease: glucocorticoids, their placental metabolism and fetal “programming” of adult pathophysiology. Nat Clin Pract Endocrinol Metab (2007) 3:479–88 10.1038/ncpendmet0515 17515892

[27] Dobbing J The later growth of the brain and its vulnerability. Pediatrics (1974) 53:2–6 4588131

[28] Giedd JN Snell JW Lange N Rajapakse JC Casey BJ Kozuch PL Quantitative magnetic resonance imaging of human brain development: ages 4-18. Cereb Cortex (1996) 6:551–60 10.1093/cercor/6.4.551 8670681

[29] Lenroot RK Giedd JN Brain development in children and adolescents: insights from anatomical magnetic resonance imaging. Neurosci Biobehav Rev (2006) 30:718–29 10.1016/j.neubiorev.2006.06.001 16887188

[30] Tau GZ Peterson BS Normal development of brain circuits. Neuropsychopharmacology (2010) 35:147–68 10.1038/npp.2009.115 19794405 PMC3055433

[31] De Bellis MD Keshavan MS Clark DB Casey BJ Giedd JN Boring AM A.E. Bennett Research Award. Developmental traumatology. Part II: brain development. Biol Psychiatry (1999) 45:1271–84 10.1016/S0006-3223(99)00045-1 10349033

[32] Suchecki D Nelson DY van Oers H Levine S Activation and inhibition of the hypothalamic-pituitary-adrenal axis of the neonatal rat: effects of maternal deprivation. Psychoneuroendocrinology (1995) 20:169–82 10.1016/0306-4530(94)00051-B 7899536

[33] van Oers HJ de Kloet ER Whelan T Levine S Maternal deprivation effect on the infant’s neural stress markers is reversed by tactile stimulation and feeding but not by suppressing corticosterone. J Neurosci (1998) 18:10171–9 9822770 10.1523/JNEUROSCI.18-23-10171.1998 PMC6793306

[34] Sapolsky RM Meaney MJ Maturation of the adrenocortical stress response: neuroendocrine control mechanisms and the stress hyporesponsive period. Brain Res (1986) 396:64–76 10.1016/0165-0173(86)90010-X 3011218

[35] Ivy AS Brunson KL Sandman C Baram TZ Dysfunctional nurturing behavior in rat dams with limited access to nesting material: a clinically relevant model for early-life stress. Neuroscience (2008) 154:1132–42 10.1016/j.neuroscience.2008.04.019 18501521 PMC2517119

[36] Hofer MA Maternal separation affects infant rats’ behavior. Behav Biol (1973) 9:629–33 10.1016/S0091-6773(73)80057-4 4761069

[37] Meaney MJ Aitken DH The effects of early postnatal handling on hippocampal glucocorticoid receptor concentrations: temporal parameters. Brain Res (1985) 22:301–4 10.1016/0165-3806(85)90183-X 4052820

[38] Meaney MJ Diorio J Francis D Widdowson J Laplante P Caldji C Early environmental regulation of forebrain glucocorticoid receptor gene expression: implications for adrenocortical responses to stress. Dev Neurosci (1996) 18:49–72 10.1159/000111395 8840086

[39] Liu D Caldji C Sharma S Plotsky PM Meaney MJ Influence of neonatal rearing conditions on stress-induced adrenocorticotropin responses and norepinepherine release in the hypothalamic paraventricular nucleus. J Neuroendocrinol (2000) 12:5–12 10.1046/j.1365-2826.2000.00422.x 10692138

[40] Viveros MP Diaz F Mateos B Rodriguez N Chowen JA Maternal deprivation induces a rapid decline in circulating leptin levels and sexually dimorphic modifications in hypothalamic trophic factors and cell turnover. Horm Behav (2010) 57:405–14 10.1016/j.yhbeh.2010.01.009 20100487

[41] Schmidt MV Enthoven L van der Mark M Levine S de Kloet ER Oitzl MS The postnatal development of the hypothalamic-pituitary-adrenal axis in the mouse. Int J Dev Neurosci (2003) 21:125–32 10.1016/S0736-5748(03)00030-3 12711350

[42] Schmidt M Enthoven L van Woezik JH Levine S de Kloet ER Oitzl MS The dynamics of the hypothalamic-pituitary-adrenal axis during maternal deprivation. J Neuroendocrinol (2004) 16:52–7 10.1111/j.1365-2826.2004.01123.x 14962076

[43] Schmidt MV Levine S Alam S Harbich D Sterlemann V Ganea K Metabolic signals modulate hypothalamic-pituitary-adrenal axis activation during maternal separation of the neonatal mouse. J Neuroendocrinol (2006) 18:865–74 10.1111/j.1365-2826.2006.01482.x 17026536

[44] Viveros MP Llorente R Lopez-Gallardo M Suarez J Bermudez-Silva F De la Fuente M Sex-dependent alterations in response to maternal deprivation in rats. Psychoneuroendocrinology (2009) 34(Suppl 1):S217–26 10.1016/j.psyneuen.2009.05.015 19553026

[45] Rice CJ Sandman CA Lenjavi MR Baram TZ A novel mouse model for acute and long-lasting consequences of early life stress. Endocrinology (2008) 149:4892–900 10.1210/en.2008-0633 18566122 PMC2582918

[46] Baram TZ Davis EP Obenaus A Sandman CA Small SL Solodkin A Fragmentation and unpredictability of early-life experience in mental disorders. Am J Psychiatry (2012) 169:907–15 10.1176/appi.ajp.2012.11091347 22885631 PMC3483144

[47] Conti G Hansman C Heckman JJ Novak MF Ruggiero A Suomi SJ Primate evidence on the late health effects of early-life adversity. Proc Natl Acad Sci U S A (2012) 109:8866–71 10.1073/pnas.1205340109 22615410 PMC3384158

[48] Kaufman D Banerji MA Shorman I Smith ELP Coplan JD Rosenblum LA Early-life stress and the development of obesity and insulin resistance in juvenile bonnet macaques. Diabetes (2007) 56:1382–6 10.2337/db06-1409 17470564

[49] Cirulli F Berry A Alleva E Early disruption of the mother-infant relationship: effects on brain plasticity and implications for psychopathology. Neurosci Biobehav Rev (2003) 27:73–82 10.1016/S0149-7634(03)00010-1 12732224

[50] D’Argenio A Mazzi C Pecchioli L Di Lorenzo G Siracusano A Troisi A Early trauma and adult obesity: is psychological dysfunction the mediating mechanism? Physiol Behav (2009) 98:543–6 10.1016/j.physbeh.2009.08.010 19733190

[51] Gunstad J Paul RH Spitznagel MB Cohen RA Williams LM Kohn M Exposure to early life trauma is associated with adult obesity. Psychiatry Res (2006) 142:31–7 10.1016/j.psychres.2005.11.007 16713630

[52] Ward AM Syddall HE Wood PJ Chrousos GP Phillips DI Fetal programming of the hypothalamic-pituitary-adrenal (HPA) axis: low birth weight and central HPA regulation. J Clin Endocrinol Metab (2004) 89:1227–33 10.1210/jc.2003-030978 15001615

[53] Wust S Entringer S Federenko IS Schlotz W Hellhammer DH Birth weight is associated with salivary cortisol responses to psychosocial stress in adult life. Psychoneuroendocrinology (2005) 30:591–8 10.1016/j.psyneuen.2005.01.008 15808929

[54] Miller AL Clifford C Sturza J Rosenblum K Vazquez DM Kaciroti N Blunted cortisol response to stress is associated with higher body mass index in low-income preschool-aged children. Psychoneuroendocrinology (2013) 38:2611–7 10.1016/j.psyneuen.2013.06.014 23849598 PMC3818281

[55] Mi J Law C Zhang KL Osmond C Stein C Barker D Effects of infant birthweight and maternal body mass index in pregnancy on components of the insulin resistance syndrome in China. Ann Intern Med (2000) 132:253–60 10.7326/0003-4819-132-4-200002150-00002 10681279

[56] Class QA Lichtenstein P Langstrom N D’Onofrio BM Timing of prenatal maternal exposure to severe life events and adverse pregnancy outcomes: a population study of 2.6 million pregnancies. Psychosom Med (2011) 73:234–41 10.1097/PSY.0b013e31820a62ce 21321257 PMC3070756

[57] Rondo PH Ferreira RF Nogueira F Ribeiro MC Lobert H Artes R Maternal psychological stress and distress as predictors of low birth weight, prematurity and intrauterine growth retardation. Eur J Clin Nutr (2003) 57:266–72 10.1038/sj.ejcn.1601526 12571658

[58] Wainstock T Anteby E Glasser S Shoham-Vardi I Lerner-Geva L The association between prenatal maternal objective stress, perceived stress, preterm birth and low birthweight. J Matern Fetal Neonatal Med (2013) 26:973–7 10.3109/14767058.2013.766696 23339660

[59] Kajantie E Fetal origins of stress-related adult disease. Ann N Y Acad Sci (2006) 1083:11–27 10.1196/annals.1367.026 17148730

[60] Li J Olsen J Vestergaard M Obel C Kristensen JK Virk J Prenatal exposure to bereavement and type-2 diabetes: a Danish longitudinal population based study. PLoS One (2012) 7:e43508 10.1371/journal.pone.0043508 22952698 PMC3429491

[61] Feldman PJ Dunkel-Schetter C Sandman CA Wadhwa PD Maternal social support predicts birth weight and fetal growth in human pregnancy. Psychosom Med (2000) 62:715–25 11020102 10.1097/00006842-200009000-00016

[62] Entringer S Wüst S Kumsta R Layes IM Nelson EL Hellhammer DH Prenatal psychosocial stress exposure is associated with insulin resistance in young adults. Am J Obstet Gynecol (2008) 199:498.e1–7 10.1016/j.ajog.2008.03.006 18448080 PMC3587039

[63] Ravelli AC van der Meulen JH Michels RP Osmond C Barker DJ Hales CN Glucose tolerance in adults after prenatal exposure to famine. Lancet (1998) 351:173–7 10.1016/S0140-6736(05)79096-6 9449872

[64] van Abeelen AFM Elias SG Bossuyt PMM Grobbee DE van der Schouw YT Roseboom TJ Famine exposure in the young and the risk of type 2 diabetes in adulthood. Diabetes (2012) 61:2255–60 10.2337/db11-1559 22648386 PMC3425424

[65] Zheng X Wang Y Ren W Luo R Zhang S Zhang JH Risk of metabolic syndrome in adults exposed to the great Chinese famine during the fetal life and early childhood. Eur J Clin Nutr (2012) 66:231–6 10.1038/ejcn.2011.161 21970943

[66] Hult M Tornhammar P Ueda P Chima C Bonamy AK Ozumba B Hypertension, diabetes and overweight: looming legacies of the Biafran famine. PLoS One (2010) 5:e13582 10.1371/journal.pone.0013582 21042579 PMC2962634

[67] Flory JD Bierer LM Yehuda R Maternal exposure to the holocaust and health complaints in offspring. Dis Markers (2011) 30:133–9 10.3233/DMA-2011-0748 21508517 PMC3825248

[68] Dancause KN Laplante DP Fraser S Brunet A Ciampi A Schmitz N Prenatal exposure to a natural disaster increases risk for obesity in 5(1/2)-year-old children. Pediatr Res (2012) 71:126–31 10.1038/pr.2011.18 22289861

[69] Dancause KN Veru F Andersen RE Laplante DP King S Prenatal stress due to a natural disaster predicts insulin secretion in adolescence. Early Hum Dev (2013) 89:773–6 10.1016/j.earlhumdev.2013.06.006 23830724 PMC3855052

[70] Ravelli AC van der Meulen JH Osmond C Barker DJ Bleker OP Obesity at the age of 50 y in men and women exposed to famine prenatally. Am J Clin Nutr (1999) 70:811–6 10539740 10.1093/ajcn/70.5.811

[71] Watt TT Appel L Roberts K Flores B Morris S Sugar, stress, and the supplemental nutrition assistance program: early childhood obesity risks among a clinic-based sample of low-income Hispanics. J Community Health (2013) 38:513–20 10.1007/s10900-012-9641-1 23197136

[72] Ertel KA Koenen KC Rich-Edwards JW Gillman MW Antenatal and postpartum depressive symptoms are differentially associated with early childhood weight and adiposity. Paediatr Perinat Epidemiol (2010) 24:179–89 10.1111/j.1365-3016.2010.01098.x 20415775 PMC4106300

[73] Alciati A Gesuele F Casazza G Foschi D The relationship between childhood parental loss and metabolic syndrome in obese subjects. Stress Health (2013) 29:5–13 10.1002/smi.1435 22190357

[74] Gundersen C Lohman BJ Garasky S Stewart S Eisenmann J Food security, maternal stressors, and overweight among low-income US children: results from the National Health and Nutrition Examination Survey (1999-2002). Pediatrics (2008) 122:e529–40 10.1542/peds.2008-0556 18762488

[75] Rich-Edwards JW Spiegelman D Lividoti Hibert EN Jun HJ Todd TJ Kawachi I Abuse in childhood and adolescence as a predictor of type 2 diabetes in adult women. Am J Prev Med (2010) 39:529–36 10.1016/j.amepre.2010.09.007 21084073 PMC3003936

[76] Danese A Pariante CM Caspi A Taylor A Poulton R Childhood maltreatment predicts adult inflammation in a life-course study. Proc Natl Acad Sci U S A (2007) 104:1319–24 10.1073/pnas.0610362104 17229839 PMC1783123

[77] Midei AJ Matthews KA Chang YF Bromberger JT Childhood physical abuse is associated with incident metabolic syndrome in mid-life women. Health Psychol (2013) 32:121–7 10.1037/a0027891 22775234 PMC3641896

[78] Lissau I Sorensen TI Parental neglect during childhood and increased risk of obesity in young adulthood. Lancet (1994) 343:324–7 10.1016/S0140-6736(94)91163-0 7905145

[79] Thomas C Hypponen E Power C Obesity and type 2 diabetes risk in midadult life: the role of childhood adversity. Pediatrics (2008) 121:e1240–9 10.1542/peds.2007-2403 18450866

[80] Reynolds RM Walker BR Syddall HE Andrew R Wood PJ Whorwood CB Altered control of cortisol secretion in adult men with low birth weight and cardiovascular risk factors. J Clin Endocrinol Metab (2001) 86:245–50 10.1210/jc.86.1.245 11232008

[81] Kant GJ Eggleston T Landman-Roberts L Kenion CC Driver GC Meyerhoff JL Habituation to repeated stress is stressor specific. Pharmacol Biochem Behav (1985) 22:631–4 10.1016/0091-3057(85)90286-2 2986182

[82] Chrousos GP Stressors, stress, and neuroendocrine integration of the adaptive response. The 1997 Hans Selye Memorial Lecture. Ann N Y Acad Sci (1998) 851:311–35 10.1111/j.1749-6632.1998.tb09006.x 9668623

[83] Grissom N Bhatnagar S Habituation to repeated stress: get used to it. Neurobiol Learn Mem (2009) 92:215–24 10.1016/j.nlm.2008.07.001 18667167 PMC2773683

[84] Daskalakis NP Bagot RC Parker KJ Vinkers CH de Kloet ER The three-hit concept of vulnerability and resilience: toward understanding adaptation to early-life adversity outcome. Psychoneuroendocrinology (2013) 38:1858–73 10.1016/j.psyneuen.2013.06.008 23838101 PMC3773020

[85] Gluckman PD Hanson MA Beedle AS Early life events and their consequences for later disease: a life history and evolutionary perspective. Am J Hum Biol (2007) 19:1–19 10.1002/ajhb.20590 17160980

[86] McEwen BS Stress, adaptation, and disease. Allostasis and allostatic load. Ann N Y Acad Sci (1998) 840:33–44 10.1111/j.1749-6632.1998.tb09546.x 9629234

[87] Schmidt MV Animal models for depression and the mismatch hypothesis of disease. Psychoneuroendocrinology (2011) 36:330–8 10.1016/j.psyneuen.2010.07.001 20674180

[88] Ng DM Jeffery RW Relationships between perceived stress and health behaviors in a sample of working adults. Health Psychol (2003) 22:638–42 10.1037/0278-6133.22.6.638 14640862

[89] Keyes KM Hatzenbuehler ML Grant BF Hasin DS Stress and alcohol: epidemiologic evidence. Alcohol Res (2012) 34:391–400 23584105 10.35946/arcr.v34.4.03 PMC3797525

[90] Sinha R Chronic stress, drug use, and vulnerability to addiction. Ann N Y Acad Sci (2008) 1141:105–30 10.1196/annals.1441.030 18991954 PMC2732004

[91] Stults-Kolehmainen MA Sinha R The effects of stress on physical activity and exercise. Sports Med (2014) 44:81–121 10.1007/s40279-013-0090-5 24030837 PMC3894304

[92] Brown R Ogden J Children’s eating attitudes and behaviour: a study of the modelling and control theories of parental influence. Health Educ Res (2004) 19:261–71 10.1093/her/cyg040 15140846

[93] Ornelas IJ Perreira KM Ayala GX Parental influences on adolescent physical activity: a longitudinal study. Int J Behav Nutr Phys Act (2007) 4:3 10.1186/1479-5868-4-3 17274822 PMC1805507

[94] Hesketh KR Goodfellow L Ekelund U McMinn AM Godfrey KM Inskip HM Activity levels in mothers and their preschool children. Pediatrics (2014) 133:e973–80 10.1542/peds.2013-3153 24664097

[95] Lussana F Painter RC Ocke MC Buller HR Bossuyt PM Roseboom TJ Prenatal exposure to the Dutch famine is associated with a preference for fatty foods and a more atherogenic lipid profile. Am J Clin Nutr (2008) 88:1648–52 10.3945/ajcn.2008.26140 19064527

[96] te Velde SJ Twisk JW van Mechelen W Kemper HC A birth-weight questionnaire indicated that life style modifies the birth weight and metabolic syndrome relationship at age 36. J Clin Epidemiol (2005) 58:1172–9 10.1016/j.jclinepi.2005.03.013 16223661

[97] Ridgway CL Brage S Anderssen SA Sardinha LB Andersen LB Ekelund U Do physical activity and aerobic fitness moderate the association between birth weight and metabolic risk in youth? The European Youth Heart Study. Diabetes Care (2011) 34:187–92 10.2337/dc10-1178 20921217 PMC3005472

[98] Kaseva N Wehkalampi K Strang-Karlsson S Salonen M Pesonen AK Raikkonen K Lower conditioning leisure-time physical activity in young adults born preterm at very low birth weight. PLoS One (2012) 7:e32430 10.1371/journal.pone.0032430 22384247 PMC3288099

[99] Falk B Eliakim A Dotan R Liebermann DG Regev R Bar-Or O Birth weight and physical ability in 5- to 8-yr-old healthy children born prematurely. Med Sci Sports Exerc (1997) 29:1124–30 10.1097/00005768-199709000-00002 9309621

[100] Nemet D Dolfin T Litmanowitz I Shainkin-Kestenbaum R Lis M Eliakim A Evidence for exercise-induced bone formation in premature infants. Int J Sports Med (2002) 23:82–5 10.1055/s-2002-20134 11842353

[101] Franks PW Ekelund U Brage S Wong MY Wareham NJ Does the association of habitual physical activity with the metabolic syndrome differ by level of cardiorespiratory fitness? Diabetes Care (2004) 27:1187–93 10.2337/diacare.27.5.1187 15111543

[102] Ortega FB Ruiz JR Hurtig-Wennlof A Meirhaeghe A Gonzalez-Gross M Moreno LA Physical activity attenuates the effect of low birth weight on insulin resistance in adolescents: findings from two observational studies. Diabetes (2011) 60:2295–9 10.2337/db10-1670 21752955 PMC3161315

[103] Aoyama T Tsushita K Miyatake N Numata T Miyachi M Tabata I Does cardiorespiratory fitness modify the association between birth weight and insulin resistance in adult life? PLoS One (2013) 8:e73967 10.1371/journal.pone.0073967 24069257 PMC3775791

[104] Volkow ND Wise RA How can drug addiction help us understand obesity? Nat Neurosci (2005) 8:555–60 10.1038/nn1452 15856062

[105] Mason SM Flint AJ Field AE Austin SB Rich-Edwards JW Abuse victimization in childhood or adolescence and risk of food addiction in adult women. Obesity (Silver Spring) (2013) 21:E775–81 10.1002/oby.20500 23637085 PMC3855159

[106] Parks EP Kumanyika S Moore RH Stettler N Wrotniak BH Kazak A Influence of stress in parents on child obesity and related behaviors. Pediatrics (2012) 130:e1096–104 10.1542/peds.2012-0895 23090343 PMC3483892

[107] Dallman MF Pecoraro NC La Fleur SE Chronic stress and comfort foods: self-medication and abdominal obesity. Brain Behav Immun (2005) 19:275–80 10.1016/j.bbi.2004.11.004 15944067

[108] Gibson EL Emotional influences on food choice: sensory, physiological and psychological pathways. Physiol Behav (2006) 89:53–61 10.1016/j.physbeh.2006.01.024 16545403

[109] Adam TC Epel ES Stress, eating and the reward system. Physiol Behav (2007) 91:449–58 10.1016/j.physbeh.2007.04.011 17543357

[110] Martikainen S Pesonen AK Lahti J Heinonen K Feldt K Pyhala R Higher levels of physical activity are associated with lower hypothalamic-pituitary-adrenocortical axis reactivity to psychosocial stress in children. J Clin Endocrinol Metab (2013) 98:E619–27 10.1210/jc.2012-3745 23471978

[111] Matthews KA Chang YF Thurston RC Bromberger JT Child abuse is related to inflammation in mid-life women: role of obesity. Brain Behav Immun (2014) 36:29–34 10.1016/j.bbi.2013.09.013 24076375 PMC3947183

[112] Gleeson M Bishop NC Stensel DJ Lindley MR Mastana SS Nimmo MA The anti-inflammatory effects of exercise: mechanisms and implications for the prevention and treatment of disease. Nat Rev Immunol (2011) 11:607–15 10.1038/nri3041 21818123

[113] Koenig JI Elmer GI Shepard PD Lee PR Mayo C Joy B Prenatal exposure to a repeated variable stress paradigm elicits behavioral and neuroendocrinological changes in the adult offspring: potential relevance to schizophrenia. Behav Brain Res (2005) 156:251–61 10.1016/j.bbr.2004.05.030 15582111

[114] Tamashiro KL Terrillion CE Hyun J Koenig JI Moran TH Prenatal stress or high-fat diet increases susceptibility to diet-induced obesity in rat offspring. Diabetes (2009) 58:1116–25 10.2337/db08-1129 19188431 PMC2671057

[115] Bian XP Seidler FJ Slotkin TA Promotional role for glucocorticoids in the development of intracellular signalling: enhanced cardiac and renal adenylate cyclase reactivity to beta-adrenergic and non-adrenergic stimuli after low-dose fetal dexamethasone exposure. J Dev Physiol (1992) 17:289–97 1337750

[116] Fowden AL Endocrine regulation of fetal growth. Reprod Fertil Dev (1995) 7:351–63 10.1071/RD9950351 8606944

[117] Cottrell EC Holmes MC Livingstone DE Kenyon CJ Seckl JR Reconciling the nutritional and glucocorticoid hypotheses of fetal programming. FASEB J (2012) 26:1866–74 10.1096/fj.12-203489 22321728

[118] Duthie L Reynolds RM Changes in the maternal hypothalamic-pituitary-adrenal axis in pregnancy and postpartum: influences on maternal and fetal outcomes. Neuroendocrinology (2013) 98:106–15 10.1159/000354702 23969897

[119] Seckl JR Prenatal glucocorticoids and long-term programming. Eur J Endocrinol (2004) 151(Suppl 3):U49–62 10.1530/eje.0.151U049 15554887

[120] Lesage J Blondeau B Grino M Breant B Dupouy JP Maternal undernutrition during late gestation induces fetal overexposure to glucocorticoids and intrauterine growth retardation, and disturbs the hypothalamo-pituitary adrenal axis in the newborn rat. Endocrinology (2001) 142:1692–702 10.1210/endo.142.5.8139 11316731

[121] Paternain L Batlle MA De la Garza AL Milagro FI Martinez JA Campion J Transcriptomic and epigenetic changes in the hypothalamus are involved in an increased susceptibility to a high-fat-sucrose diet in prenatally stressed female rats. Neuroendocrinology (2012) 96:249–60 10.1159/000341684 22986707

[122] Nyirenda MJ Lindsay RS Kenyon CJ Burchell A Seckl JR Glucocorticoid exposure in late gestation permanently programs rat hepatic phosphoenolpyruvate carboxykinase and glucocorticoid receptor expression and causes glucose intolerance in adult offspring. J Clin Invest (1998) 101:2174–81 10.1172/JCI1567 9593773 PMC508805

[123] Cleasby ME Livingstone DE Nyirenda MJ Seckl JR Walker BR Is programming of glucocorticoid receptor expression by prenatal dexamethasone in the rat secondary to metabolic derangement in adulthood? Eur J Endocrinol (2003) 148:129–38 10.1530/eje.0.1480129 12534366

[124] Jones AP Simson EL Friedman MI Gestational undernutrition and the development of obesity in rats. J Nutr (1984) 114:1484–92 6540299 10.1093/jn/114.8.1484

[125] Bertram CE Hanson MA Animal models and programming of the metabolic syndrome. Br Med Bull (2001) 60:103–21 10.1093/bmb/60.1.103 11809621

[126] Remacle C Bieswal F Bol V Reusens B Developmental programming of adult obesity and cardiovascular disease in rodents by maternal nutrition imbalance. Am J Clin Nutr (2011) 94:1846S–52S 10.3945/ajcn.110.001651 21543546

[127] Vickers MH Gluckman PD Coveny AH Hofman PL Cutfield WS Gertler A Neonatal leptin treatment reverses developmental programming. Endocrinology (2005) 146:4211–6 10.1210/en.2005-0581 16020474

[128] Vickers MH Breier BH McCarthy D Gluckman PD Sedentary behavior during postnatal life is determined by the prenatal environment and exacerbated by postnatal hypercaloric nutrition. Am J Physiol Regul Integr Comp Physiol (2003) 285:R271–3 12794001 10.1152/ajpregu.00051.2003

[129] Llorente-Berzal A Fuentes S Gagliano H Lopez-Gallardo M Armario A Viveros MP Sex-dependent effects of maternal deprivation and adolescent cannabinoid treatment on adult rat behaviour. Addict Biol (2011) 16:624–37 10.1111/j.1369-1600.2011.00318.x 21521421

[130] Mela V Llorente-Berzal A Diaz F Argente J Viveros MP Chowen JA Maternal deprivation exacerbates the response to a high fat diet in a sexually dimorphic manner. PLoS One (2012) 7(11):e48915 10.1371/journal.pone.0048915 23145019 PMC3492147

[131] Maniam J Morris MJ Palatable cafeteria diet ameliorates anxiety and depression-like symptoms following an adverse early environment. Psychoneuroendocrinology (2010) 35:717–28 10.1016/j.psyneuen.2009.10.013 19939573

[132] Maniam J Morris MJ Voluntary exercise and palatable high-fat diet both improve behavioural profile and stress responses in male rats exposed to early life stress: role of hippocampus. Psychoneuroendocrinology (2010) 35:1553–64 10.1016/j.psyneuen.2010.05.012 20594764

[133] Machado TD Molle RD Laureano DP Portella AK Werlang ICR Benetti CD Early life stress is associated with anxiety, increased stress responsivity and preference for “comfort foods” in adult female rats. Stress (2013) 16:549–56 10.3109/10253890.2013.816841 23781957

[134] Bernardi JR Ferreira CF Senter G Krolow R de Aguiar BW Portella AK Early life stress interacts with the diet deficiency of omega-3 fatty acids during the life course increasing the metabolic vulnerability in adult rats. PLoS One (2013) 8(4):e62031 10.1371/journal.pone.0062031 23614006 PMC3629088

[135] Ryu V Yoo SB Kang DW Lee JH Jahng JW Post-weaning isolation promotes food intake and body weight gain in rats that experienced neonatal maternal separation. Brain Res (2009) 1295:127–34 10.1016/j.brainres.2009.08.006 19666012

[136] Miki T Liu JQ Ohta K Suzuki S Kusaka T Warita K Early postnatal maternal separation causes alterations in the expression of beta3-adrenergic receptor in rat adipose tissue suggesting long-term influence on obesity. Biochem Biophys Res Commun (2013) 442:68–71 10.1016/j.bbrc.2013.11.005 24220331

[137] Avishai-Eliner S Gilles EE Eghbal-Ahmadi M Bar-El Y Baram TZ Altered regulation of gene and protein expression of hypothalamic-pituitary-adrenal axis components in an immature rat model of chronic stress. J Neuroendocrinol (2001) 13:799–807 10.1046/j.1365-2826.2001.00698.x 11578530 PMC3100736

[138] Pihoker C Owens MJ Kuhn CM Schanberg SM Nemeroff CB Maternal separation in neonatal rats elicits activation of the hypothalamic-pituitary-adrenocortical axis: a putative role for corticotropin-releasing factor. Psychoneuroendocrinology (1993) 18:485–93 10.1016/0306-4530(93)90042-J 8265736

[139] Nilsson C Jennische E Ho HP Eriksson E Bjorntorp P Holmang A Increased insulin sensitivity and decreased body weight in female rats after postnatal corticosterone exposure. Eur J Endocrinol (2002) 146:847–54 10.1530/eje.0.1460847 12039706

[140] Shpilberg Y Beaudry JL D’Souza A Campbell JE Peckett A Riddell MC A rodent model of rapid-onset diabetes induced by glucocorticoids and high-fat feeding. Dis Model Mech (2012) 5:671–80 10.1242/dmm.008912 22184636 PMC3424464

[141] D’Souza AM Beaudry JL Szigiato AA Trumble SJ Snook LA Bonen A Consumption of a high-fat diet rapidly exacerbates the development of fatty liver disease that occurs with chronically elevated glucocorticoids. Am J Physiol Gastrointest Liver Physiol (2012) 302:G850–63 10.1152/ajpgi.00378.2011 22268100

[142] Taskinen MR Nikkila EA Pelkonen R Sane T Plasma lipoproteins, lipolytic enzymes, and very low density lipoprotein triglyceride turnover in Cushing’s syndrome. J Clin Endocrinol Metab (1983) 57:619–26 10.1210/jcem-57-3-619 6348067

[143] Wajchenberg BL Subcutaneous and visceral adipose tissue: their relation to the metabolic syndrome. Endocr Rev (2000) 21:697–738 10.1210/edrv.21.6.0415 11133069

[144] Rockall AG Sohaib SA Evans D Kaltsas G Isidori AM Monson JP Computed tomography assessment of fat distribution in male and female patients with Cushing’s syndrome. Eur J Endocrinol (2003) 149:561–7 10.1530/eje.0.1490561 14640998

[145] Buren J Liu HX Jensen J Eriksson JW Dexamethasone impairs insulin signalling and glucose transport by depletion of insulin receptor substrate-1, phosphatidylinositol 3-kinase and protein kinase B in primary cultured rat adipocytes. Eur J Endocrinol (2002) 146:419–29 10.1530/eje.0.1460419 11888850

[146] Schacke H Docke WD Asadullah K Mechanisms involved in the side effects of glucocorticoids. Pharmacol Ther (2002) 96:23–43 10.1016/S0163-7258(02)00297-8 12441176

[147] Bernal-Mizrachi C Weng S Feng C Finck BN Knutsen RH Leone TC Dexamethasone induction of hypertension and diabetes is PPAR-alpha dependent in LDL receptor-null mice. Nat Med (2003) 9:1069–75 10.1038/nm898 12847522

[148] Reynolds RM Walker BR Human insulin resistance: the role of glucocorticoids. Diabetes Obes Metab (2003) 5:5–12 10.1046/j.1463-1326.2003.00221.x 12542720

[149] Kuo T Lew MJ Mayba O Harris CA Speed TP Wang JC Genome-wide analysis of glucocorticoid receptor-binding sites in myotubes identifies gene networks modulating insulin signaling. Proc Natl Acad Sci U S A (2012) 109:11160–5 10.1073/pnas.1111334109 22733784 PMC3396543

[150] Hajduch E Hainault I Meunier C Jardel C Hainque B Guerre-Millo M Regulation of glucose transporters in cultured rat adipocytes: synergistic effect of insulin and dexamethasone on GLUT4 gene expression through promoter activation. Endocrinology (1995) 136:4782–9 10.1210/endo.136.11.7588207 7588207

[151] Dimitriadis G Leighton B Parry-Billings M Sasson S Young M Krause U Effects of glucocorticoid excess on the sensitivity of glucose transport and metabolism to insulin in rat skeletal muscle. Biochem J (1997) 321(Pt 3):707–12 9032457 10.1042/bj3210707 PMC1218126

[152] Weinstein SP Wilson CM Pritsker A Cushman SW Dexamethasone inhibits insulin-stimulated recruitment of GLUT4 to the cell surface in rat skeletal muscle. Metabolism (1998) 47:3–6 10.1016/S0026-0495(98)90184-6 9440469

[153] Moisan MP Seckl JR Edwards CR 11 Beta-hydroxysteroid dehydrogenase bioactivity and messenger RNA expression in rat forebrain: localization in hypothalamus, hippocampus, and cortex. Endocrinology (1990) 127:1450–5 10.1210/endo-127-3-1450 2387261

[154] Whorwood CB Donovan SJ Flanagan D Phillips DI Byrne CD Increased glucocorticoid receptor expression in human skeletal muscle cells may contribute to the pathogenesis of the metabolic syndrome. Diabetes (2002) 51:1066–75 10.2337/diabetes.51.4.1066 11916927

[155] Yehuda R Seckl J Minireview: stress-related psychiatric disorders with low cortisol levels: a metabolic hypothesis. Endocrinology (2011) 152:4496–503 10.1210/en.2011-1218 21971152

[156] Jamieson PM Chapman KE Edwards CR Seckl JR 11 Beta-hydroxysteroid dehydrogenase is an exclusive 11 beta- reductase in primary cultures of rat hepatocytes: effect of physicochemical and hormonal manipulations. Endocrinology (1995) 136:4754–61 10.1210/en.136.11.4754 7588203

[157] Gathercole LL Lavery GG Morgan SA Cooper MS Sinclair AJ Tomlinson JW 11Beta-hydroxysteroid dehydrogenase 1: translational and therapeutic aspects. Endocr Rev (2013) 34:525–55 10.1210/er.2012-1050 23612224

[158] Tomlinson JW Walker EA Bujalska IJ Draper N Lavery GG Cooper MS 11β-Hydroxysteroid dehydrogenase type 1: a tissue-specific regulator of glucocorticoid response. Endocr Rev (2004) 25:831–66 10.1210/er.2003-0031 15466942

[159] Cleasby ME Kelly PA Walker BR Seckl JR Programming of rat muscle and fat metabolism by in utero overexposure to glucocorticoids. Endocrinology (2003) 144:999–1007 10.1210/en.2002-220559 12586777

[160] Masuzaki H Flier JS Tissue-specific glucocorticoid reactivating enzyme, 11 beta-hydroxysteroid dehydrogenase type 1 (11 beta-HSD1) – a promising drug target for the treatment of metabolic syndrome. Curr Drug Targets Immune Endocr Metabol Disord (2003) 3:255–62 10.2174/1568008033340135 14683456

[161] Amatruda JM Danahy SA Chang CL The effects of glucocorticoids on insulin-stimulated lipogenesis in primary cultures of rat hepatocytes. Biochem J (1983) 212:135–41 6347191 10.1042/bj2120135 PMC1152020

[162] Berdanier CD Role of glucocorticoids in the regulation of lipogenesis. FASEB J (1989) 3:2179–83 2666232 10.1096/fasebj.3.10.2666232

[163] Noguchi T Iritani N Tanaka T Molecular mechanism of induction of key enzymes related to lipogenesis. Proc Soc Exp Biol Med (1992) 200:206–9 10.3181/00379727-200-43419 1579584

[164] Vasiljevic A Velickovic N Bursac B Djordjevic A Milutinovic DV Nestorovic N Enhanced prereceptor glucocorticoid metabolism and lipogenesis impair insulin signaling in the liver of fructose-fed rats. J Nutr Biochem (2013) 24:1790–7 10.1016/j.jnutbio.2013.04.001 23773625

[165] Dowman JK Hopkins LJ Reynolds GM Armstrong MJ Nasiri M Nikolaou N Loss of 5alpha-reductase type 1 accelerates the development of hepatic steatosis but protects against hepatocellular carcinoma in male mice. Endocrinology (2013) 154(12):4536–47 10.1210/en.2013-1592 24080367 PMC4192287

[166] Paterson JM Morton NM Fievet C Kenyon CJ Holmes MC Staels B Metabolic syndrome without obesity: hepatic overexpression of 11beta-hydroxysteroid dehydrogenase type 1 in transgenic mice. Proc Natl Acad Sci U S A (2004) 101:7088–93 10.1073/pnas.0305524101 15118095 PMC406470

[167] Paterson JM Holmes MC Kenyon CJ Carter R Mullins JJ Seckl JR Liver-selective transgene rescue of hypothalamic-pituitary-adrenal axis dysfunction in 11beta-hydroxysteroid dehydrogenase type 1-deficient mice. Endocrinology (2007) 148:961–6 10.1210/en.2006-0603 17170103 PMC6443039

[168] Carter RN Paterson JM Tworowska U Stenvers DJ Mullins JJ Seckl JR Hypothalamic-pituitary-adrenal axis abnormalities in response to deletion of 11beta-HSD1 is strain-dependent. J Neuroendocrinol (2009) 21:879–87 10.1111/j.1365-2826.2009.01899.x 19602102 PMC2810446

[169] Chapman K Holmes M Seckl J 11Beta-hydroxysteroid dehydrogenases: intracellular gate-keepers of tissue glucocorticoid action. Physiol Rev (2013) 93:1139–206 10.1152/physrev.00020.2012 23899562 PMC3962546

[170] Paternain L Martisova E Milagro FI Ramirez MJ Martinez JA Campion J Postnatal maternal separation modifies the response to an obesogenic diet in adulthood in rats. Dis Model Mech (2012) 5:691–7 10.1242/dmm.009043 22773756 PMC3424467

[171] Nyirenda MJ Carter R Tang JI de Vries A Schlumbohm C Hillier SG Prenatal programming of metabolic syndrome in the common marmoset is associated with increased expression of 11beta-hydroxysteroid dehydrogenase type 1. Diabetes (2009) 58:2873–9 10.2337/db09-0873 19720800 PMC2780883

[172] Gustafsson JA Stenberg A Irreversible androgenic programming at birth of microsomal and soluble rat liver enzymes active on androstene-3,17-dione and 5alpha-androstane-3alpha,17beta-diol. J Biol Chem (1974) 249:711–8 4811898

[173] Stimson RH Lobley GE Maraki I Morton NM Andrew R Walker BR Effects of proportions of dietary macronutrients on glucocorticoid metabolism in diet-induced obesity in rats. PLoS One (2010) 5:e8779 10.1371/journal.pone.0008779 20098742 PMC2808251

[174] Drake AJ Raubenheimer PJ Kerrigan D McInnes KJ Seckl JR Walker BR Prenatal dexamethasone programs expression of genes in liver and adipose tissue and increased hepatic lipid accumulation but not obesity on a high-fat diet. Endocrinology (2010) 151:1581–7 10.1210/en.2009-1088 20133452

[175] Chen N Liu L Zhang Y Ginsberg HN Yu Y-H Whole-body insulin resistance in the absence of obesity in FVB mice with overexpression of Dgat1 in adipose tissue. Diabetes (2005) 54:3379–86 10.2337/diabetes.54.12.3379 16306352

[176] Bruce KD Cagampang FR Argenton M Zhang J Ethirajan PL Burdge GC Maternal high-fat feeding primes steatohepatitis in adult mice offspring, involving mitochondrial dysfunction and altered lipogenesis gene expression. Hepatology (2009) 50:1796–808 10.1002/hep.23205 19816994

[177] Lehmann J Feldon J Long-term biobehavioral effects of maternal separation in the rat: consistent or confusing? Rev Neurosci (2000) 11:383–408 10.1515/REVNEURO.2000.11.4.383 11065281

[178] Lippmann M Bress A Nemeroff CB Plotsky PM Monteggia LM Long-term behavioural and molecular alterations associated with maternal separation in rats. Eur J Neurosci (2007) 25:3091–8 10.1111/j.1460-9568.2007.05522.x 17561822

[179] Macri S Chiarotti F Wurbel H Maternal separation and maternal care act independently on the development of HPA responses in male rats. Behav Brain Res (2008) 191:227–34 10.1016/j.bbr.2008.03.031 18468700

[180] Wortwein G Husum H Andersson W Bolwig TG Mathe AA Effects of maternal separation on neuropetide Y and calcitonin gene-related peptide in “depressed” Flinders sensitive line rats: a study of gene-environment interactions. Prog Neuropsychopharmacol Biol Psychiatry (2006) 30:684–93 10.1016/j.pnpbp.2006.01.027 16600456

[181] Krysiak R Obuchowicz E Herman Z Interactions between the neuropeptide Y system and the hypothalamic-pituitary-adrenal axis. Eur J Endocrinol (1999) 140:130–6 10.1530/eje.0.1400130 10069655

[182] Maniam J Morris MJ The link between stress and feeding behaviour. Neuropharmacology (2012) 63:97–110 10.1016/j.neuropharm.2012.04.017 22710442

[183] Cavagnini F Croci M Putignano P Petroni ML Invitti C Glucocorticoids and neuroendocrine function. Int J Obes Relat Metab Disord (2000) 24(Suppl 2):S77–9 10.1038/sj.ijo.0801284 10997615

[184] Liu D Diorio J Day JC Francis DD Meaney MJ Maternal care, hippocampal synaptogenesis and cognitive development in rats. Nat Neurosci (2000) 3:799–806 10.1038/77702 10903573

[185] Kauffman AS Coming of age in the kisspeptin era: sex differences, development, and puberty. Mol Cell Endocrinol (2010) 324:51–63 10.1016/j.mce.2010.01.017 20083160 PMC2902563

